# GBA1 inactivation in oligodendrocytes affects myelination and induces neurodegenerative hallmarks and lipid dyshomeostasis in mice

**DOI:** 10.1186/s13024-024-00713-z

**Published:** 2024-03-07

**Authors:** Ilaria Gregorio, Loris Russo, Enrica Torretta, Pietro Barbacini, Gabriella Contarini, Giada Pacinelli, Dario Bizzotto, Manuela Moriggi, Paola Braghetta, Francesco Papaleo, Cecilia Gelfi, Enrico Moro, Matilde Cescon

**Affiliations:** 1 Department of Molecular Medicine, University of Padova, Via Ugo Bassi 58/B, 35131 Padua, Italy; 2 Laboratory of Proteomics and Lipidomics, IRCCS Orthopedic Institute Galeazzi, Milan, 20161 Italy; 3 Department of Biomedical Sciences for Health, University of Milan, 20133 Milan, Italy; 4 Genetics of Cognition Laboratory, Neuroscience Area, Istituto Italiano Di Tecnologia, 16163 Genova, Italy; 5 Department of Biomedical and Technological Sciences, University of Catania, 95125 Catania, Italy; 6 Padova Neuroscience Center (PNC), University of Padova, 35131 Padua, Italy

**Keywords:** Parkinson’s disease, Gaucher disease, Oligodendrocyte, White matter, β-glucocerebrosidase, Myelination, Lipid dyshomeostasis, Neurodegeneration

## Abstract

**Background:**

Mutations in the β-glucocerebrosidase (*GBA1*) gene do cause the lysosomal storage Gaucher disease (GD) and are among the most frequent genetic risk factors for Parkinson’s disease (PD). So far, studies on both neuronopathic GD and PD primarily focused on neuronal manifestations, besides the evaluation of microglial and astrocyte implication. White matter alterations were described in the central nervous system of paediatric type 1 GD patients and were suggested to sustain or even play a role in the PD process, although the contribution of oligodendrocytes has been so far scarcely investigated.

**Methods:**

We exploited a system to study the induction of central myelination in vitro, consisting of Oli-neu cells treated with dibutyryl-cAMP, in order to evaluate the expression levels and function of β-glucocerebrosidase during oligodendrocyte differentiation. Conduritol-B-epoxide, a β-glucocerebrosidase irreversible inhibitor was used to dissect the impact of β-glucocerebrosidase inactivation in the process of myelination, lysosomal degradation and α-synuclein accumulation in vitro. Moreover, to study the role of β-glucocerebrosidase in the white matter in vivo, we developed a novel mouse transgenic line in which β-glucocerebrosidase function is abolished in myelinating glia, by crossing the *Cnp1-cre* mouse line with a line bearing *loxP* sequences flanking *Gba1* exons 9–11, encoding for β-glucocerebrosidase catalytic domain. Immunofluorescence, western blot and lipidomic analyses were performed in brain samples from wild-type and knockout animals in order to assess the impact of genetic inactivation of β-glucocerebrosidase on myelination and on the onset of early neurodegenerative hallmarks, together with differentiation analysis in primary oligodendrocyte cultures.

**Results:**

Here we show that β-glucocerebrosidase inactivation in oligodendrocytes induces lysosomal dysfunction and inhibits myelination in vitro. Moreover, oligodendrocyte-specific β-glucocerebrosidase loss-of-function was sufficient to induce in vivo demyelination and early neurodegenerative hallmarks, including axonal degeneration, α-synuclein accumulation and astrogliosis, together with brain lipid dyshomeostasis and functional impairment.

**Conclusions:**

Our study sheds light on the contribution of oligodendrocytes in GBA1-related diseases and supports the need for better characterizing oligodendrocytes as actors playing a role in neurodegenerative diseases, also pointing at them as potential novel targets to set a brake to disease progression.

**Supplementary Information:**

The online version contains supplementary material available at 10.1186/s13024-024-00713-z.

## Background

β-glucocerebrosidase is a membrane-bound lysosomal enzyme involved in the degradation of glycosphingolipids, including glucosylceramide (GlcCer) and glucosylsphingosine (GlcSph) [1]. Mutations affecting β-glucocerebrosidase activity cause Gaucher disease (GD), a rare disorder belonging to the group of lysosomal storage diseases (LSDs). In GD, the deficient hydrolysis of β-glucocerebrosidase substrates and their subsequent progressive accumulation, especially in macrophages, leads to clinical manifestations affecting several organs and resulting in hepatosplenomegaly, bone defects, anaemia, and lung disease [2, 3]. While less prominent in type 1 GD, neurological manifestations occur in the neuronopathic forms of GD (type 2 and 3) and can include seizure, cognitive impairment, motor and coordination deficit, and psychiatric disorders [4, 5]. These symptoms have been linked to β-glucocerebrosidase substrates accumulation occurring in neurons [6, 7], triggering neurotoxicity, astrogliosis and neuroinflammation in both human patients [8] and mouse models [9, 10]. *GBA1* mutations are also the most common genetic risk factor for Parkinson’s disease (PD), increasing the risk of PD onset by 20 to 30 times [11]. About 20% of adult GD patients, including type 1 patients, develop Parkinson’s related symptoms upon reaching adulthood [12, 13].

In GBA1-related diseases, an impairment in lysosomal function due to β-glucocerebrosidase loss-of-function is considered a common pathological mechanism [14]. In both GD and GBA1-related PD, defects in the autophagy-lysosomal system have been shown to be critical for neurodegeneration [15]. The accumulation of undegraded autophagic and β-glucocerebrosidase substrates accompanies with α-synuclein accrual in the brain of genetic or chemically-induced animal models of PD lacking a functional β-glucocerebrosidase, as well as in cell cultures derived from PD patients [16–19]. Interestingly, GlcCer was shown to stabilise oligomeric α-synuclein and, in a sort of positive feedback loop, α-synuclein was able to inhibit β-glucocerebrosidase activity, leading to a worsening of the disease [20].

Of note, most of the in vivo and in vitro studies focusing on shared mechanisms between GD and GBA1-related PD target dopaminergic neurons, the most affected neuronal population in PD [21]. However, depleting β-glucocerebrosidase only in dopaminergic neurons does not induce marked neurodegeneration [22], whereas PD human specimens clearly show the involvement of glial cells in the pathology [8, 23]. Recent experimental studies provided proofs for the involvement of glial β-glucocerebrosidase in GD and GBA1-related PD: specific β-glucocerebrosidase depletion in astrocytes and microglia causes neuroinflammation, lysosomal defects, α-synuclein accumulation and altered crosstalk with neurons [24–28].

Oligodendrocytes, the myelin-producing glia of the central nervous system (CNS), were far less investigated in the context of GBA1-related disorders although clinical studies provided a rationale to assess their involvement. Myelin is a lipid-rich stack of insulating membranes that allows saltatory conduction of action potentials and protects axons [29]. Due to the biochemical composition of myelin sheaths, oligodendrocytes require a finely balanced lipid anabolism and catabolism for their synthesis and maintenance [30] as well as channels for axo-glial exchange of nutrients and organelles [31] to the cell periphery and axons. Interestingly, GD patients display, at clinical evaluation, white matter (WM) abnormalities both in the neuronopathic and non-neuronopathic forms of the disease [32–34], and WM involvement was also reported in PD with relevance for motor performance [35].

Our study shows that oligodendrocyte differentiation and myelination require β-glucocerebrosidase activity in vitro and in vivo. Both chemically and genetically induced β-glucocerebrosidase loss-of-function in oligodendrocytes inhibits myelination and turns out into lysosomal dysfunction leading to α-synuclein accumulation and altered lipid profile. Our in vivo studies demonstrate that oligodendrocyte defects are able to elicit neurodegenerative hallmarks already at 6 months of age, including axonal defects, microglial activation and astrogliosis, as well as behavioural deficits in mice. These findings point at a major contribution of oligodendroglial cells to neurodegeneration upon loss of β-glucocerebrosidase function, therefore highlighting the relevance of oligodendrocyte dysfunction in the onset and/or progression of GBA1-related neuropathologies.

## Methods

### Mice and genotyping

Procedures on mice were approved by the Ethics Committee of the University of Padova and authorised by the Italian Ministry of Health. Heterozygous *Gba1*^*f/*+^ mice [36] were kindly provided by Dr. Anthony Futerman (Weizmann Institute of Science, Israel) on behalf of Dr. Stefan Karlsson (Lund University, Sweden), while *Cnp1*^*wt/cre*^ mice [37] were kindly provided by Dr. Armin Klaus Nave (Max Planck Institute for Multidisciplinary Sciences, Göttingen, Germany). Heterozygous *Gba1*^*f/*+^ were crossed to obtain *Gba1*^*f/f*^. These mice were then crossed with *Cnp1*^*wt/cre*^ to generate *Gba1*^*f/f*^*:: Cnp1*^*wt/cre*^ mice (hereon called *Gba1*^*f/f*^*::cre*). Oligodendrocyte specific *Gba1* knockout mice littermates lacking the cre transgene (named *Gba1*^*f/f*^) were used as control. The genotype was determined through PCR analysis on DNA extracted from tail or ear biopsies. The tail was digested overnight at 56° C in a Tris–HCl and SDS buffer with proteinase K. DNA was then purified with isopropanol precipitation and a wash in ethanol. For ear tissue, samples were incubated for 1 h at 90° C in a 50 mM NaOH solution, which was then neutralized with a Tris–HCl buffer. The primers used for the genotyping are shown in Supplementary Table 1. Male and female mice were sacrificed by cervical dislocation. During the molecular characterization no differences were seen between male and female *Gba1*^*f/f*^*::cre* compared to their siblings, therefore both sexes were analyzed and data were plotted together except when indicated. Behavioral tests were performed blindly, without knowing genotypes. 

### Behavioral tests

#### Gait analysis

Locomotor abilities of mice were assessed by analyzing the gait [38]. Before starting the test, mice were allowed to get familiar with test procedures. The hindlimbs and forelimbs of mice were stained with non-toxic paint, and each animal was allowed to walk along 1 m-long and 5 cm-wide strips of paper in a wooden corridor closed on its sides. Stride length and base width were measured.

#### Buried pellet

Olfactory capacity was tested with the buried pellet test. Mice were starved on the night prior to the test day. Each mouse was placed in a clean cage with a 4–5 cm deep layer of fresh sawdust. A food pellet was placed 0.5 cm beneath the sawdust and the mouse was then allowed to walk freely inside of the cage. The task was considered ended as soon as the mouse found the food and showed interest. Each mouse was tested twice with the food pellet placed in a different place inside the cage. The two performance times were averaged and used for statistical analysis.

#### Rotarod

Balance and motor coordination was tested on the rotarod. Mice were trained the day before the test by performing three trials at a speed of 4 rpm, with a 1-min pause between each trial. The trial ended if the mouse fell or after 5 min on the rotarod. On the day of the final test, mice were tested three times with a 10-min interval between trials. The rotarod was set to start at a speed of 4 rpm for 1 min, then the speed was set to ramp up to 40 rpm in 5 min. The test ended when the mouse fell or after 5 min.

#### Locomotor activity

Mice were tested for 55 min in an experimental apparatus consisting of two open-field arenas (38 × 51 × 30 cm), evenly illuminated by overhead white lighting (25 ± 5 lx). Each test was recorded by using a digital video camera and analyzed with ANY-maze tracking software system (Stoelting). In order to assess any possible motor alterations (e.g., hyperlocomotion), we examined locomotor activity by quantifying the distance travelled (m) in the empty arena per 5 min epochs. Furthermore, time spent (s) within the internal/external zone was determined.

### Culture of Oli-neu cell line

Oligodendrocyte cell line Oli-neu [39] was kindly provided by Dr. Patrizia Rosa (CNR-Institute of Neuroscience, Milan) on behalf of Dr. Jacqueline Trotter (University of Mainz, Mainz, Germany). Cells were cultured at 37° C at 5% CO_2_ on high molecular weight poly-L-lysine (Merck) coated flasks in SATO medium, prepared by adding the following reagents to high-glucose DMEM (Gibco) according to [40]: 23.8 µM NaHCO_3_ (Merck), 1.25 mM Apo-transferrin (Merck), 1.72 µM Insulin (Merck), 0.1 mM Putrescine (Merck), 0.2 µM Progesterone (Merck), 0.5 mM Triiodo-L-Thyronine (Merck), 0.22 µM Sodium selenite (Merck), 0.5 µM L-Thyroxine (Merck), 52.3 µM Gentamicin (Thermo Fisher Scientific), Horse Serum 1% (Gibco). The culture medium was replaced every 48 h during expansion. Cells were detached by using 0.01% trypsin–EDTA at 60–70% confluency for further expansion or plating. The differentiation into mature oligodendroglial cells was induced by treating Oli-neu cells with 1 mM dibutyryl-cyclic AMP (dbcAMP, Merck) for up to 72 h. Conduritol B epoxide (CBE, Merck) was added in the medium at a concentration of 10 µM to inhibit β-glucocerebrosidase activity. In some experiments cells were plated on glass coverslips for imaging and plastic wells for RNA and protein extraction.

### Isolation and culture of primary oligodendrocytes

Primary oligodendrocyte cultures were obtained by adapting the protocol from [41]. Newborn 2-days-old mice derived from crossing *Gba1*^*f/f*^ with *Gba1*^*f/f*^*::cre* mice were sacrificed and heads were sterilised with 70% ethanol. The skin was cut and the skull was carefully detached from the underlying tissue. The whole brain was then placed in ice-cold HBSS (Gibco) supplemented with 2% Pen/Strep (Gibco). Cortexes were dissected and placed in HBSS with Pen/Strep in a 10 mm petri dish, then transferred to a laminar flow hood and mechanically homogenized to roughly 1 mm^3^ pieces with a sterile scalpel blade after HBSS removal. The pieces were then further homogenized by pipetting them through a 5 ml serological pipette containing DMEM/F-12 (Gibco) supplemented with 20% FBS (Gibco), 2% Pen/Strep and 1% Amphotericin B (Gibco). Preparations were then cultured in T25 cell culture flasks for 10 days to allow OPC proliferation on a confluent cell layer. The culture medium was replaced every 2–3 days. After 10 days in culture, microglia detachment was favored by shaking at 200 rpm at 37° C for 1 h. The supernatant was discarded, new fresh medium was added and OPCs were detached by culture shaking at 250 rpm for 20 h. OPCs were transferred to a 10 mm petri dish for 10 min, to allow the attachment of contaminant microglia and astrocytes. The floating OPCs were collected and plated on PLL-coated multi-well in proliferation medium composed of DMEM/F-12 supplemented with 1% N-2 supplement (Gibco), 2% B-27 serum free supplement (Gibco), 0.1% BSA (Merck), 2% Pen/Strep, 10 ng/ml rFGF2 (Cambridge Stem Cell Institute) and 10 ng/ml rmPDGFaa (ImmunoTools). After 2 days, proliferation medium was replaced with a differentiation medium containing TIT at a final concentration of 500 nM. The obtained primary OPCs were then differentiated for 4–6 days.

### LysoTracker staining

LysoTracker Green (DND-26, Thermo Fisher Scientific) was used to detect acidic lysosomes by confocal microscopy. Oli-neu cells were plated on PLL-coated glass coverslips in a 24-well plate. On the last day of culture, LysoTracker was added to the culture medium at 50 nM concentration and incubated for 90 min at 37° C in the dark. Cells were then washed and briefly fixed with PFA (Merck) 4% in PBS, to be immediately acquired with a confocal microscope (Zeiss LSM700) or to perform immunofluorescence. The software Fiji (https://imagej.net/software/fiji/) was used to quantify the LysoTracker-positive puncta.

### Filipin III staining for cholesterol

Staining of cholesterol in Oli-neu cells was performed with the Cholesterol Cell-Based Detection Assay Kit (Cayman Chemical). In brief, cells were plated on a 96-well plate, then on the last day of culture cells were fixed for 10 min and washed three times. Filipin III solution was prepared and added to the wells, then the plate was incubated at room temperature in the dark for 1 h. At the end of Filipin III incubation, cells were washed twice and visualized at an inverted microscope (Leica DMI4000).

### TUNEL assay

The In situ cell death detection kit TMR red (Merck) was used to perform TUNEL (TdT-mediated dUTP nick end labelling) assay of Oli-neu cells, following the manufacturer instructions. Cells were fixed with 4% PFA in PBS and permeabilized for 5 min with a PBS solution containing 0.1% Triton X-100 (Merck) and sodium citrate (J.T. Baker). rTdT enzyme solution was prepared according to the manufacturer’s instructions and incubated for 1 h at 37° C in the dark. Hoechst 33258 (Merck) was used at a concentration of 10 µg/ml to stain nuclei. Glass coverslips were mounted using 80% glycerol (Merck) in PBS and images were acquired with a fluorescence microscope (Leica DM5000B). TUNEL-positive nuclei were quantified blindly using the Fiji software on randomly acquired fields.

### β-glucocerebrosidase enzymatic activity

The activity of β-glucocerebrosidase was assayed on Oli-neu cells and mouse tissue protein extracts. For every sample, 6 µg of protein extract were mixed with dH_2_O to a final volume of 10 µl. Protein extracts were then incubated for 3 h in the dark at 37° C after adding an equal volume of methylumbelliferyl-B-D-glucopyranoside (MUG) 5 mM acetate buffer solution, pH 4.2. The enzymatic reaction was stopped by adding a carbonate buffer at pH 10.7, and samples were loaded in triplicate on a 96-well plate. The plate was analyzed with a fluorometer (Victor plate reader) which was set to an excitation wavelength of 365 nm and emission of 448 nm.

### Western blotting

Protein extracts were obtained either from Oli-neu cells, primary oligodendrocytes or tissues. Oli-neu cells and primary oligodendrocytes were washed and scraped after adding RIPA lysis buffer containing 50 mM Tris–HCl (Merck), 150 mM NaCl (Merck), 1% IgePal (Merck), 0.5% sodium deoxycholate (Merck), 0.1% SDS (Merck), supplemented with EDTA-free protease inhibitors (Roche) and phosphatase inhibitor cocktail II (Sigma-Aldrich). The same buffer was used to lyse tissues after being mechanically dissociated with a mortar and pestle. Samples were centrifuged at 4° C at 16,000 × *g* and the protein-containing supernatants were collected. Protein concentration was measured with the BCA Protein Kit Assay (Pierce). Protein samples were mixed with sample buffer and reducing agent (Thermo Fisher Scientific) and denatured at 90° C for 10 min, then loaded on a polyacrylamide gel (Thermo Fisher Scientific) for SDS-PAGE. After electrophoresis, gels were blotted onto PVDF membranes (Thermo Fisher Scientific) and blocked using either 5% non-fat dry milk or 5% BSA in 1% Tween-20 (Merck) in tris-Buffered saline (TBST). For the detection of both phosphorylated and non-phosphorylated α-synuclein membranes were subjected to fixation with 4% PFA in PBS for 30 min prior to the blocking step, to increase sensitivity for the protein [42]. Primary antibodies were diluted in 5% BSA or 2.5% milk in TBST and incubated overnight at 4° C. Membranes were washed with TBST and incubated with HRP-conjugated secondary antibodies (Amersham Bioscience) for 1 h at room temperature. The detection of proteins was achieved by incubating membranes with a chemiluminescent solution (Pierce) in a transilluminator (ImageQuant LAS4000) and analyzed with Fiji software. Primary antibodies used are shown in Supplementary Table 2.

### RNA extraction and quantitative PCR

Oli-neu cells, primary oligodendrocytes or tissue homogenates were dissolved in Trizol Reagent (Thermo Fisher Scientific) and total RNA was extracted following the manufacturer’s instructions. The extracted RNA was quantified with a NanoDrop spectrophotometer (Thermo Fisher Scientific), and reverse transcription was carried out using Moloney Murine Leukemia Virus Reverse Transcriptase (M-MLV RT, Invitrogen). The obtained cDNA was then analyzed by real-time quantitative PCR with the Solis BioDyne mastermix on a Rotorgene (Qiagen). The sequence of primers used to amplify the cDNA are reported in Supplementary Table 3.

### Immunofluorescence

Immunofluorescence was performed on Oli-neu and primary oligodendrocytes plated on glass coverslips. On the last day of culture, cells were washed and fixed with 4% PFA in PBS for 10 min. After washing with PBS, Oli-neu were permeabilized for 5 min with a solution of 0.5% Triton X-100 in PBS, while 0.2% PBS-Tween was used for primary oligodendrocytes. Unspecific bindings were blocked with 10% goat serum in PBS for 1 h at room temperature. The blocking solution was discarded, and cells were incubated with primary antibodies in 5% goat serum in PBS overnight at 4° C. Cells were washed in PBS and incubated with secondary antibodies in 5% goat serum in PBS for 1 h at RT. For GlcCer detection, the permeabilization step was reduced to 2 min to preserve lipidic antigens. For immunofluorescence on tissue samples, pieces were frozen in liquid N_2_ and 10-μm slices were cut with a cryostat at -20 °C, then dried overnight at RT. Glass slides were washed in PBS and fixed/permeabilized in a 1:1 solution of methanol-acetone for 10 min at -20° C. Glass slides were washed and incubated with a blocking solution containing 10% goat serum in PBS for 1 h at room temperature. Slides were then incubated overnight with the primary antibodies at 4° C. On the following day, glass slides were washed and incubated with secondary antibodies for 1 h at room temperature. When primary antibodies produced in mouse were used, slices were blocked for 2.5 h with a solution containing 10% IgG free BSA. Then, mouse IgG in the tissue were masked by incubating the slices with a goat anti-mouse IgG antibody (Jackson ImmunoResearch) for 30 min at room temperature. For the detection of IBA1, tissue slices were fixed for 30 min with 4% PFA in PBS. For detection of α-syn aggregation, tissue slices were fixed with 4% PFA in PBS, for 15 min. Then, samples were incubated with a blocking solution containing 10% goat serum and 0.3% Triton X-100 in PBS for 2 h at room temperature. To improve the detection of myelin with antibodies or FluoroMyelin™ Green (Thermo Fisher Scientific, F34651), samples were fixed for 1 h with 4% PFA in PBS, then permeabilized in 100% ethanol at -20° C for 12 min [43]. Glass slides were then mounted with 80% glycerol in PBS and images were acquired using confocal (Zeiss LSM700, Leica Stellaris 8) or fluorescent microscopes (Leica DM5000B). Immunofluorescence intensity was quantified on unprocessed original images. Fluorescence intensity was normalized by the number of nuclei for Oli-neu cells and by area in tissue slices, after selection of the region of interest (ROI).

### Transmission electron microscopy

Striatum and optic nerves dissected from each mouse were fixed overnight in a 0.1 M cacodylate buffer containing 2% PFA and 2.5% glutaraldehyde. The samples were then placed in cacodylate buffer and processed for electron microscopy. Briefly, tissues were embedded in epoxide resin and ultrathin sections were stained with uranyl acetate and lead citrate. Slices were acquired with a FET Tecnai 12 transmission electron microscope (Electron Microscopy Service, Biology Department, University of Padova). The g-ratio and degenerating axon analyses were performed on randomly selected fields by using the software Fiji (https://imagej.net/software/fiji/) for quantification.

### NF-L ELISA assay

Mice at the age of 6 months were sacrificed by cervical dislocation and blood was collected in a 1.5 ml Eppendorf tube. Blood samples were allowed to clot for 2 h then centrifuged for 15 min at 1000 rcf. The supernatant (serum) was collected and stored at -80° C until the assay was performed. The mouse NF-L ELISA assay (NBP2-80,299 Novus Biologicals) was performed following the manufacturer’s instructions. Briefly, for each animal 100 µl of serum were loaded in duplicate and allowed to incubate for 90 min at 37° C. Serum samples were then removed, and the biotinylated detection antibody solution was added and incubated for 1 h at 37° C. The wells were washed three times, then the HRP conjugate was added and incubated for 30 min at 37° C. After 5 washes, 90 µl of substrate reagent were added to each well and incubated for 30 min at 37° C. Finally, 50 µl of stop solution were added and the sample absorbance was measured at 450 nm. The serum concentration of NF-L was calculated by a four-parameter logistic (4PL) curve based on the absorbance of serial dilutions of NF-L provided in the kit. Sample values were corrected by subtracting the blank absorbance.

### Lipidomic analysis

Oli-neu cells (1 × 10^6^ cells) were resuspended in 100 μl of PBS 1 × and sonicated. After the addition of 100ul of ultrapure water and 1.5 ml of methanol/chloroform 2:1 containing 0.01% w/v BHT, samples were placed in a water bath at 37° C overnight under constant stirring. 150 μl of 1 M KOH in methanol were added and vials were placed at 37° C for two hours. Saponification was stopped by addition of 150 μl of 1 M acetic acid. After drying under nitrogen stream, samples were resuspended in 150 μl of methanol and centrifuged at 10,000 × *g* for 3 min. After phase separation, organic phases were collected in 2 ml glass vials, dried and resuspended in 50 μl of methanol. 10 μl of sphingolipid extract were loaded in duplicate and subsequently developed on a 20 × 10 cm HPTLC plate with silica surface (Merck) using Linomat 5 semiautomatic TLC spotter (CAMAG, Switzerland) and developed in 55:20:3 chloroform/methanol/water (v/v/v) using a Twin Trough Chamber (Camag). After drying, plate was sprayed with fluorescent dye primuline (5% w/v in 80:20 acetone/water (v/v)) and scanned with Ettan Dige Imager (EDI; Molecular Dynamics) with the lamp set at 480 excitation wavelength with a 530 filter for emission. Band quantification was performed using Image Quant TL software (Molecular Dynamics) normalizing against the total amount of detected sphingolipids. Hexosylsphingosine (HexSph), hexosylceramides (HexCers) and hexosylcholesterol were quantified by targeted mass spectrometry (MRM-MS) using a Xevo TQ-S micro mass spectrometer (Waters, Milford, MA, USA). Oli-neu cells were resuspended in PBS, sonicated and total protein content determined by BCA Protein Assay (Thermo Fisher Scientific). A volume corresponding to 100 µg of protein was fortified with 2 pmol of lactosylsphingosine (LysoGb2) and 200 pmol of glucosyl (β) ceramide (d18:1/12:0). After the addition of 1.5 ml of 2:1 methanol/chloroform containing 0.01% w/v BHT, samples were heated at 48 °C overnight. Then, 150 µl of 1 M KOH in methanol were added and, after a 2-h incubation at 37 °C, the solution was neutralized with 150 µl of 1 M acetic acid and dried with Speedvac. Samples were then respunded in 0.15 ml of methanol, and centrifuged for 3 min at 10,000 × g. Liquid phases were collected in UPLC glass vials and stored at -80 °C. 10 µl of extract were injected and separated on a C8 Acquity UPLC BEH (Waters, Milford, MA, USA), 100 mm × 2.1 mm id, 1.7 µm, kept at 30 °C, using the following linear gradient: 0.0 min: 80% B; 3 min: 90% B; 6 min: 90% B; 15 min: 99% B; 18 min: 99% B; 20 min: 80% B, at 0.3 ml/min flow rate. Phase B consisted of 1 mM ammonium formate in methanol, 0.05 mM formic acid, while phase A was 2 mM ammonium formate in H_2_O with 0.05 mM formic acid. An electrospray interface operating in positive ion mode was employed to obtain MS/MS spectra by acquiring MRM transitions of hexosylsphingosine (HexSph), hexosylceramides (HexCers) and hexosylcholesterol (HexChol) indicated in Supplementary Table 4. The capillary voltage was set at 3.5 kV. The source temperature was set to 150 °C. The desolvation gas flow was set to 1,000, and the desolvation temperature was set to 350 °C. Data were acquired by MassLynx™ 4.2 software and quantified by TargetLynx software.

Brains from 6-month-old male mice were harvested and immediately frozen in liquid N_2_. 30 mg of tissue were mixed with 750 uL of 0.9% NaCl w/v and sonicated. After centrifugation at 13,000 × *g* for 15 min, surnatants were recovered and total protein content determined by Pierce bicinchoninic acid (BCA) protein assay (Thermo Fisher Scientific). A volume corresponding to 500 μg of protein was transferred to a new vial and fortified with 2 pmol of lysoGb2 and 200 pmol of glucosyl (β)ceramide (d18:1/12:0), ceramide (d18:1/12:0), sphingosine (d17:1), sphingosine-1-phosphate (d17:1) and sphingomyelin (d18:1/12:0) and extracted as described for cells. Hexosylceramides, dihexosylceramides, ceramides, dihydroceramides and sphingomyelins were detected by untargeted mass spectrometry on a Q-TOF Synapt G2-Si (Waters, Milford, MA, USA). The ESI ionization source was operated in positive ion mode with 30 V of sample cone voltage and 3.0 kV of capillary voltage. The desolvation temperature was set to 150° C, and the ion source was 120° C. The desolvation gas flow was set to 600 L/h. Data were captured under centroid mode, and the range of mass scan was set to 50–1500 Da. Accuracy and reproducibility were maintained employing an independent reference spray via LockSpray. Sphingolipids’ quantification was carried out using the ion chromatogram obtained for each compound using 50 mDa windows. The linear dynamic range was determined by the injection of standard mixtures. Positive identification of compounds was based on the accurate mass measurement, with an error < 5 ppm and its retention time, compared to that of a standard (± 2%). Mass spectra were analyzed by MassLynx™ 4.2 Software (Waters, Milford, MA, USA), and lipids were annotated as lipid subclasses as follows (sphingosine backbone/number of carbon atoms of the fatty acid: number of unsaturation of the fatty acid). MS/MS spectra were acquired, and the assignment of species was based on precursor ions and product ions m/z 264.268 and m/z 266.286, which correspond to sphingosine backbone (d18:1) and dihydrosphingosine backbone (d18:0), respectively. Sphingosine, dihydrosphingosine, sphingosine-1-phosphate, dihydro-sphingosine-1-phosphate and hexosylcholesterol were quantified by MRM-MS as described above ( with MRM transitions listed in Supplementary Table 5). 

### Statistical analysis

Statistical analysis was performed using GraphPad Prism Software v.8.0.2. The statistical tests used are described in the respective figure legends. If not otherwise specified, unpaired two-tailed Student’s t-test was used to compare differences between genotypes for normally distributed data. *P* values equal or less than 0.05 were considered statistically significant. N corresponds to independent biological replicates. For in vivo studies, n corresponds to individual mice. For in vitro studies, n corresponds to individual primary oligodendrocytes cultures or Oli-neu cells at different passages.

## Results

### β-glucocerebrosidase is upregulated in oligodendroglial differentiation

**Fig. 1:**
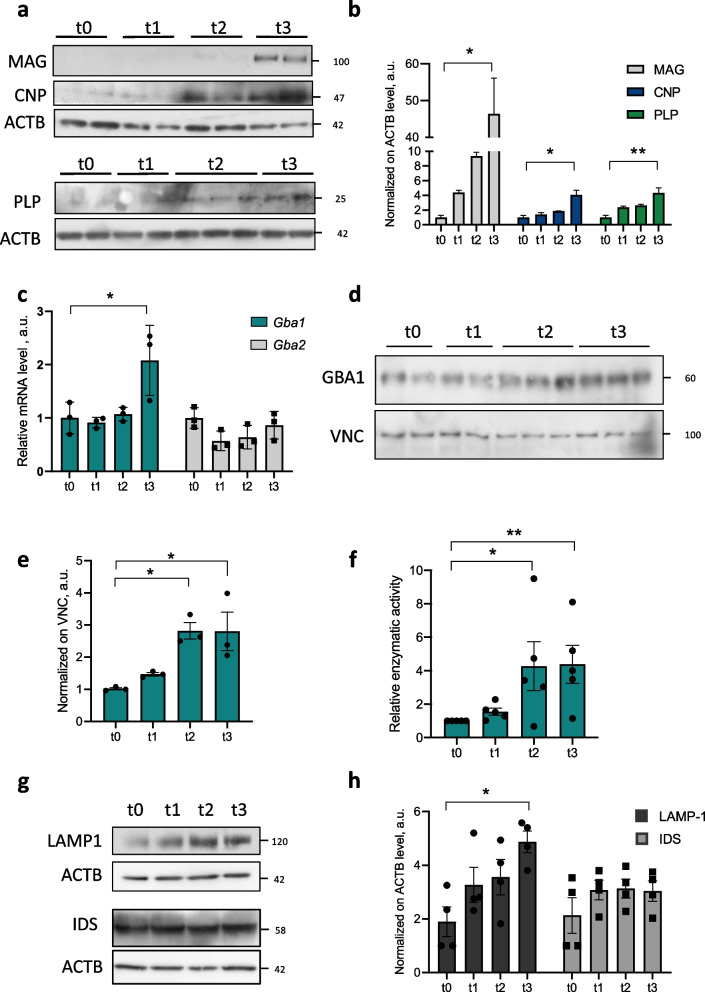
β-glucocerebrosidase is upregulated upon myelination induction in vitro. **a** Representative western blot of protein extracts from Oli-neu cells untreated or treated for 1, 2 or 3 days with dbcAMP (t0, t1, t2 and t3, respectively). MAG, CNP and PLP myelin protein levels were analyzed; β-actin (ACTB) was used as loading control. **b** Densitometric quantification of western blots as in (**a**), showing expression of CNP1, PLP and MAG myelin proteins in Oli-neu cells treated with dbcAMP (*, *p* < 0.05; Kruskal–Wallis test and Dunn's test for multiple comparison, *n* = 3 independent experiments). Error bars indicate s.e.m. **c** qRT-PCR analysis of *Gba1* and *Gba2* mRNA from Oli-neu cells treated with dbcAMP for three days. *Actb* was used as housekeeping gene (*, *p* < 0.05; Kruskal–Wallis test and Dunn's test for multiple comparison, *n* = 3 independent experiments). Error bars indicate s.e.m. **d** Representative western blot of GBA1 protein levels in Oli-neu cells upon dbcAMP treatment. Vinculin (VNC) was used as loading control. **e** Densitometric quantification of western blot as in (**d**) (*, *p < *0.05; **, *p* < 0.001; unpaired two-tailed Student’s *t* test; *n* = 3 independent experiments). Error bars indicate s.e.m. **f** Enzymatic activity assay performed on protein extracts from Oli-neu cells treated with dbcAMP (*, *p* < 0.05; **, *p* < 0.01; Kruskal–Wallis test and Dunn's test for multiple comparison; *n* = 5 independent experiments). Error bars indicate s.e.m. **g** Representative western blot of LAMP1 and IDS protein levels in Oli-neu cells treated with dbcAMP. ACTB was used as loading control. **h** Densitometric quantification of western blot as in (**g**) (*, *p* < 0.05; Kruskal–Wallis test and Dunn's test for multiple comparison, *n* = 4 independent experiments). Error bars indicate s.e.m

To investigate the relevance of β-glucocerebrosidase in oligodendrocytes, we exploited an established system to study the induction of central myelination in vitro, consisting of Oli-neu cells treated with dibutyryl-cAMP (dbcAMP) [39, 44, 45]. Besides the expected increase in myelin-related protein levels (MAG, CNP and PLP; Fig. 1a, b), Oli-neu cells treated with dbcAMP for three days showed higher gene expression for the lysosomal enzyme *Gba1*, but not for the cytosolic *Gba2,* compared to untreated cells (Fig. 1c). Such increase was also detected at the β-glucocerebrosidase protein level (Fig. 1d, e), in parallel to a raise in its enzymatic activity (Fig. 1f). In keeping with the relevance for lysosomal function in central myelin development [46, 47], we found increased LAMP1 protein levels in differentiating cells, whereas the protein levels of a different lysosomal enzyme such as iduronate 2-sulfatase (IDS) were unaffected (Fig. 1g, h). Correspondingly, neither the mRNA levels of *Ids* nor of the galactosylceramidase enzyme (*Galc)* were found upregulated (Supplementary Fig. 1a), thus pointing at a specificity for β-glucocerebrosidase upregulation upon myelination induction in vitro.

### β-glucocerebrosidase inhibition impairs the lysosomal degradative system activity

**Fig. 2:**
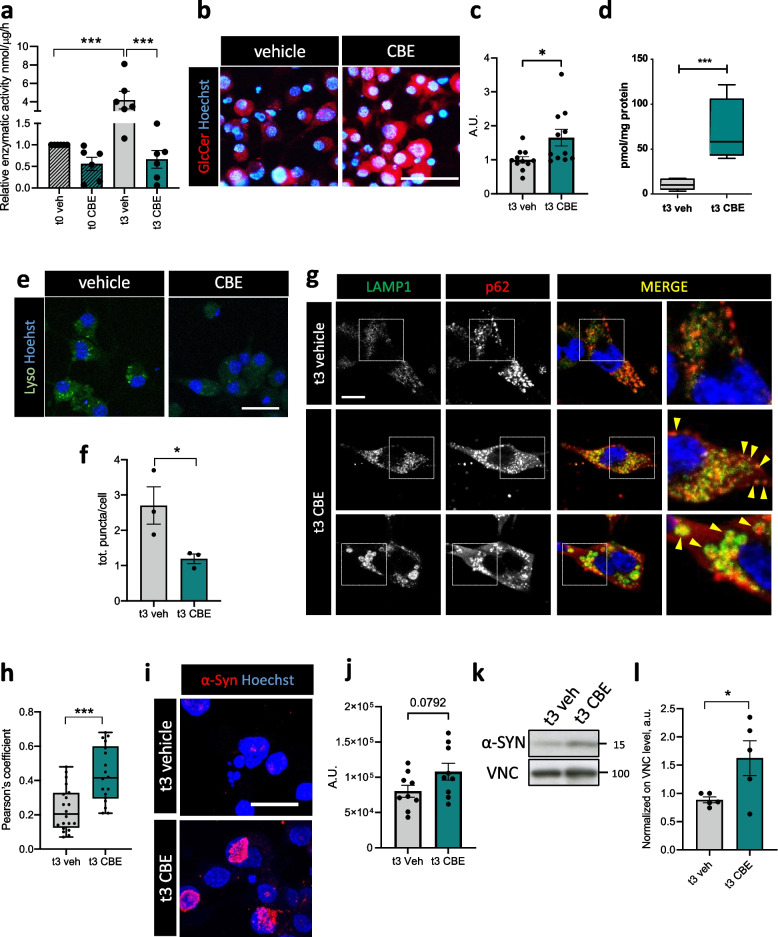
β-glucocerebrosidase inhibition induces substrate accumulation and reduced lysosomal activity leading to autophagic cargo accrual. **a** GBA1 enzymatic activity assay performed on protein extracts from undifferentiated and differentiated cells (t0 and t3) upon vehicle (veh) or CBE treatment (***, *p* < 0.001; Kruskal–Wallis test and Dunn's test for multiple comparison; *n* = 5 independent experiments). Error bars indicate s.e.m. **b** Immunofluorescence staining on differentiated Oli-neu cells upon vehicle (veh) or CBE treatment. Scale bar = 100 μm **c** Quantification of GlcCer integrated density in differentiated vehicle (veh) and CBE treated cells (*, *p* < 0.05; unpaired two-tailed Student’s t test; *n* = 11 randomly selected fields from 3 independent experiments). Error bars indicate s.e.m. **d** MRM-MS quantitation of Hexosylsphingosine (HexSph) in differentiated vehicle (veh) and CBE treated Oli-neu (***, *p* < 0.001; unpaired two-tailed Student’s t test; *n* = 4 independent experiments). The whiskers of the box plot represent the minimum and the maximum data values. **e** Representative images of LysoTracker staining of Oli-neu cells upon vehicle (veh) or CBE treatment at t3. Scale bar = 20 μm. **f** Quantification of LysoTracker-positive puncta in vehicle (veh) and CBE treated cells at t3 (*, *p* < 0.05; unpaired two-tailed Student’s t test; *n* = 3 independent experiments). Error bars indicate s.e.m. **g** Representative immunofluorescence for LAMP1 and p62 staining on Oli-neu cells upon vehicle or CBE treatment at t3. Scale bar = 20 μm. **h** Quantification of LAMP and p62 colocalization upon vehicle (veh) or CBE treatment in differentiated Oli-neu cells (***, *p* < 0.001; unpaired two-tailed Student’s t-test; *n* = 3 independent experiments). Error bars indicate s.e.m. **i** Immunofluorescence of differentiated Oli-neu cells upon vehicle (veh) or CBE treatment at t3 showing α-synuclein (α-syn) positive clusters in red. Scale bar = 50 μm. **j** Quantification of α-syn integrated density in differentiated vehicle (veh) and CBE treated cells (unpaired two-tailed Student’s t test; *n* = 9 randomly selected fields form 3 independent experiments). Error bars indicate s.e.m. **k**, **l** Western blot analysis of α-synuclein monomer (α-SYN) on differentiated Oli-neu cells treated with vehicle or CBE, and relative densitometric quantification. Vinculin (VNC) was used as loading control (*, *p* < 0.05; unpaired two-tailed Student’s t-test; *n* = 5 independent experiments). Error bars indicate s.e.m

To further assess the role of β-glucocerebrosidase function in this culture system, Oli-neu cells were treated with conduritol B epoxide (CBE), an irreversible β-glucocerebrosidase inhibitor previously used to model GD and PD in vitro and in vivo [9, 48], for three days (t3) during dbcAMP-induced differentiation. CBE treatment led to significantly reduced β-glucocerebrosidase activity at t3 (Fig. 2a), as well as to a significant accumulation of the specific β-glucocerebrosidase substrates GlcCer (Fig. 2b, c) and HexSph (Fig. 2d), while no HexChol accumulation was detected (Supplementary 1b). Interestingly, the decrease in β-glucocerebrosidase activity and the parallel increase in GlcCer levels were more evident in differentiated cells than in undifferentiated ones (Fig. 2a, b; Supplementary Fig. 1c), suggesting a more prominent role of β-glucocerebrosidase in myelinating oligodendrocytes rather than in the immature ones.

*GBA1* mutations were previously found to alter lysosomal pH, with detrimental effects on ﻿key survival processes such as macroautophagy and chaperone-mediated autophagy, in both embryonic fibroblasts and neurons [48]. To investigate whether β-glucocerebrosidase inhibition was able to alter lysosomal functionality also in differentiating Oli-neu cells, we probed lysosomal acidity by mean of LysoTracker, a lipophilic lysosomal acidic pH sensor, after confirming the proper co-localization of the probe with the lysosomal marker LAMP1 in basal conditions (Supplementary Fig. 1d). Interestingly, CBE-treated cells displayed a significantly reduced number of LysoTracker-positive puncta, when compared to vehicle treated Oli-neu cells upon differentiation (Fig. 2e, f), indicating lysosomal alkalinization, although without any significant change in total LAMP1 level (Supplementary Fig. 1e, f). CBE-treated myelinating cells showed increased co-immunolocalization between LAMP1 and p62 autophagic cargo, with higher amounts of p62 aggregates retained within enlarged lysosomes (Fig. 2g, h), pointing at a reduced capacity of degrading autophagic-related targets.

Defective protein clearance was previously accounted as a shared mechanism underlying the two major GBA1-related disorders, GD and PD, involving the lysosomal target α-synuclein, a relevant pathological marker [19, 49]. Of note, the *Snca* gene, coding for α-synuclein, although primarily expressed by neurons, is also expressed by both immature and mature oligodendrocytes [50, 51]. In agreement with an impairment of the degradative lysosomal pathway, CBE-treated differentiated cells showed accumulation of α-synuclein-positive clusters that were less present in vehicle-treated cells (Fig. 2i, j). Western blot analysis confirmed increased levels of α-synuclein upon CBE treatment (Fig. 2k, l), indicating that α-synuclein turnover is affected by β-glucocerebrosidase dysfunction in differentiated Oli-neu cells.

### β-glucocerebrosidase inhibition alters oligodendrocyte differentiation and lipid homeostasis

**Fig. 3:**
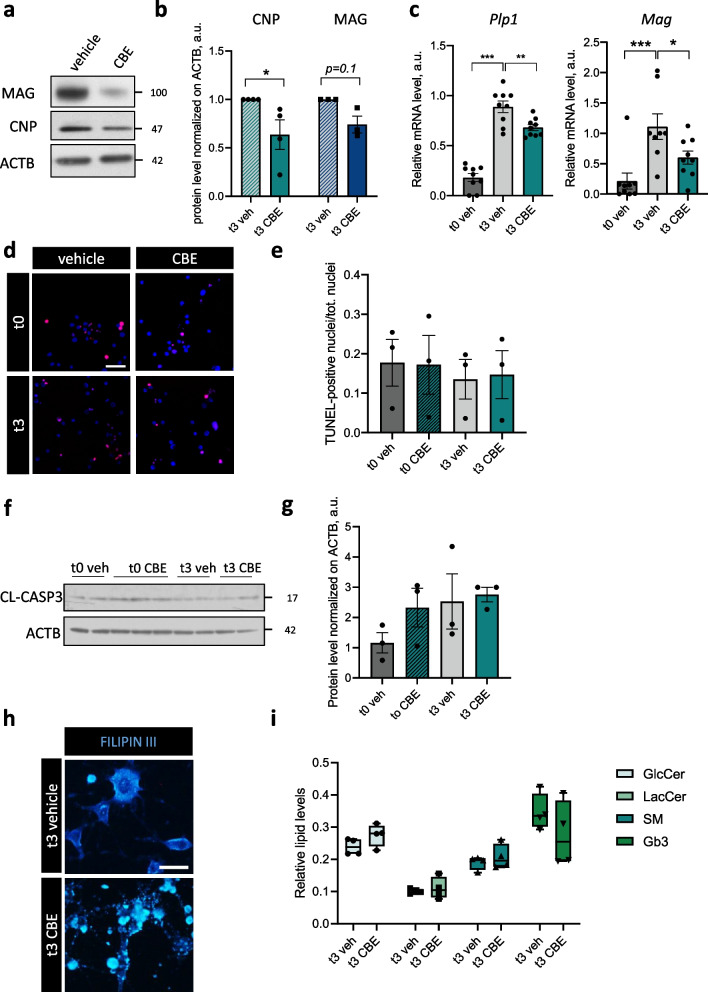
Oli-neu differentiation and lipid homeostasis is impaired upon reduction of β-glucocerebrosidase activity. **a** Representative western blot of CNP and MAG protein levels in differentiated Oli-neu cells upon vehicle (veh) and CBE treatment. β-actin (ACTB) was used as loading control. **b** Densitometric quantification of western blots for CNP and MAG upon dbcAMP treatment (t3) in the presence of vehicle (veh) or CBE (*, *p* < 0.05; unpaired two-tailed Mann–Whitney U test; CNP, *n* = 4; MAG, *n* = 3 independent experiments). Error bars indicate s.e.m. **c** qRT-PCR for *Plp1* (left panel) and *Mag* (right panel) gene expression in Oli-neu cells at t0 and t3, upon vehicle (veh) or CBE treatment (*, *p* < 0.05; **, *p* < 0.01; unpaired two-tailed Student’s *t* test; *n* = 9 samples from 3 independent experiments). Error bars indicate s.e.m. **d**,** e** Representative images of TUNEL test and quantification of TUNEL-positive nuclei normalized on the total number of cells at t0 and t3 differentiated Oli-neu treated with vehicle (veh) or CBE (Kruskal–Wallis test and Dunn's test for multiple comparison; n = 4 independent experiments). Scale bar = 100 μm. **f**,** g** Representative western blot (**f**) and densitometric quantification of cleaved caspase-3 (CL-CASP3) in t0 and t3 differentiated Oli-neu cells upon treatment with vehicle (veh) and CBE. β-actin (ACTB) was used as loading control (Kruskal–Wallis test and Dunn's test for multiple comparison; n = 3 independent experiments). Error bars indicate s.e.m. **h** Filipin III staining of differentiated Oli-neu cells treated with vehicle (veh) or CBE at t3. Scale bar = 50 μm. **i** High Performance Thin Layer Chromatography (HPTLC) analysis of glucosylceramide (GlcCer), lactosylceramide (LacCer), sphingomyelin (SM) and globotriaosylceramide (Gb3), reported as relative levels to total resolved lipids (unpaired two-tailed Student’s t-test; *n* = 4 independent experiments). Error bars indicate maximum and minimum values

Considering the upregulation of *Gba1* expression upon myelination induction, we further investigated whether CBE could alter Oli-neu differentiation. Indeed, differentiated cells showed lower levels of myelin proteins, including MAG and CNP, upon β-glucocerebrosidase inhibition (Fig. 3a, b). Such results appeared an effect of delayed myelination, since lower transcript levels of myelin genes including *Plp1* and *Mag* were observed at t3 upon β-glucocerebrosidase inhibition (Fig. 3c), while apoptosis, assessed by both TUNEL assay and cleaved caspase-3 protein levels, was not induced by CBE treatment in differentiated cells (Fig. 3d-g).

β-glucocerebrosidase defects were shown to induce global changes in cellular lipid composition [52]. Cholesterol is expected to accumulate due to β-glucocerebrosidase loss of function, pointing to a role for the enzyme not only in sphingolipid but also in steroid metabolism [53]. Accordingly, differentiated cells displayed accumulation of free cholesterol upon CBE treatment, as revealed by Filipin III staining (Fig. 3h). To better address the presence of unbalanced lipid homeostasis, we measured the levels of GlcCer, together with lactosylceramide (LacCer) and globotriaosylceramide (Gb3), as direct products of GlcCer modifications, and sphingomyelin (SM), as parallelly involved in ceramide biosynthetic pathways. Despite not significant, we observed a trend toward an increase of GlcCer and LacCer, as expected, due to β-glucocerebrosidase inactivation, but also of SM, consistent with a reduced activity of acid-sphingomyelinase in impaired lysosomes [14] (Fig. 3i).

### β-glucocerebrosidase inactivation causes demyelination in vivo

With the aim of investigating the role of β-glucocerebrosidase in WM in vivo, we developed a novel mouse transgenic line in which β-glucocerebrosidase function is abolished in myelinating glia. The *Cnp1-cre* mouse line [37] was crossed with a line bearing *loxP* sequences flanking *Gba1* exons 9–11 [36], producing a mouse line with deletion of the exons encoding for β-glucocerebrosidase catalytic domain in oligodendrocytes (and Schwann cells), namely *Gba1*^*f/f*^*::Cnp1*^*Wt/cre*^ (hereafter and in figures abbreviated as *Gba1*^*f/f*^*::cre;* Supplementary Fig. 2a, b). The abatement of the full-length product was confirmed by western blotting with an antibody recognizing the C-terminus of the enzyme, showing marked decrease of β-glucocerebrosidase protein levels in primary cultures of both oligodendrocytes and Schwann cells (Supplementary Fig. 2c, d).

In order to set our studies at a mature stage of myelination, when the reduction of β-glucocerebrosidase elicited by genetic ablation of the catalytic domain was well established (Supplementary Fig. 2e, f) and the decline of β-glucocerebrosidase enzymatic activity was detectable in different CNS areas (Supplementary Fig. 2 g), we focused on 6 months of age, as a key point to estimate the contribution of oligodendrocyte β-glucocerebrosidase loss-of-function to early neurodegeneration and/or potential accumulation of substrates.

**Fig. 4:**
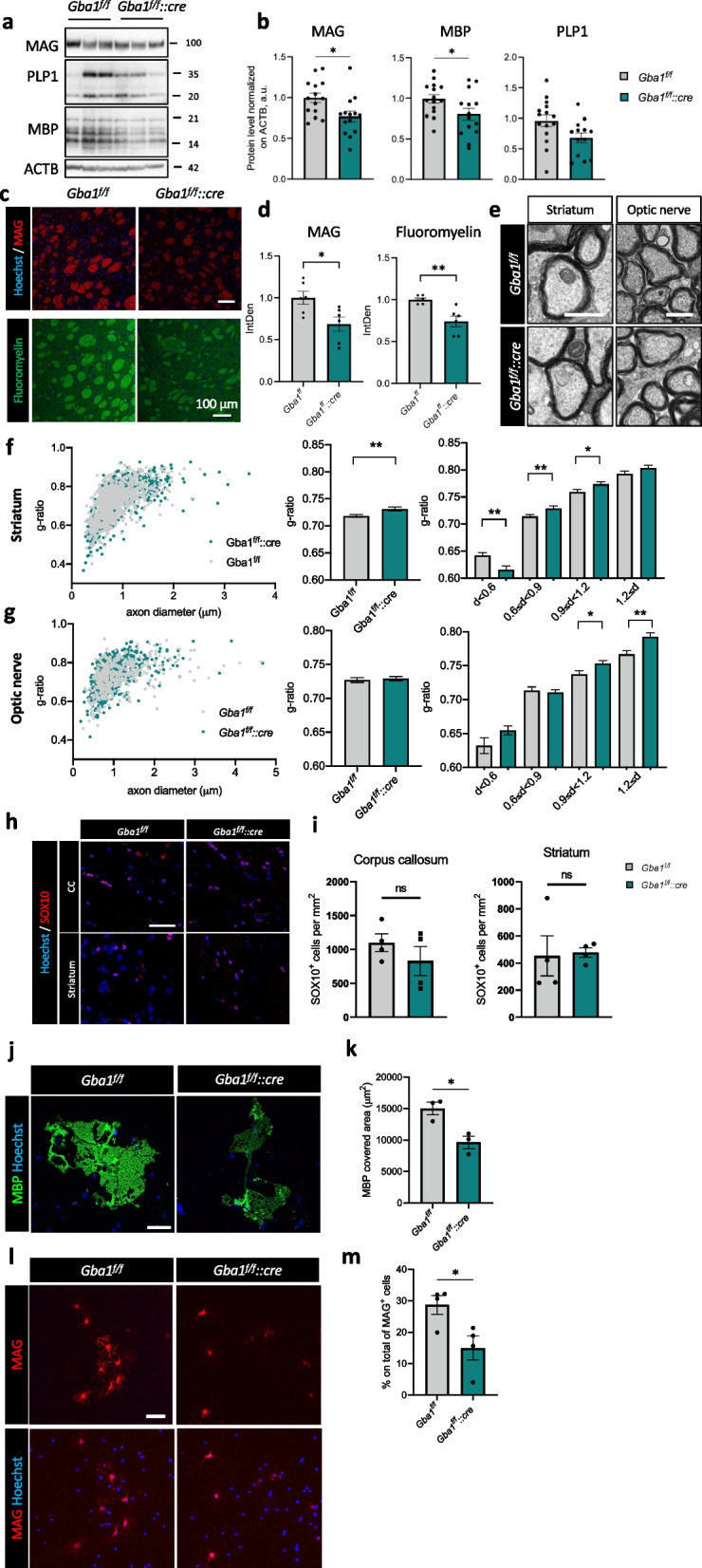
β-glucocerebrosidase inactivation in oligodendrocytes causes demyelination in adult mice and affects oligodendrocyte differentiation in vitro.** a**,** b** Representative western blot (**a**) and relative densitometric quantification (**b**) of total brain protein extracts from *Gba1*^*f/f*^ and *Gba1*^*f/f*^*::cre* mice of 6 months of age. MAG, PLP1 and MBP myelin protein levels were analyzed. β-actin (ACTB) was used as loading control (*, *p* < 0.05; unpaired two-tailed Student’s t-test; MAG, *n* = 14 mice; MBP, *n* = 15 mice for *Gba1*^*f/f*^ and *n* = 14 mice for *Gba1*^*f/f*^*::cre*; PLP1, n = 15 mice for *Gba1*^*f/f*^ and *n* = 13 mice for *Gba1*^*f/f*^*::cre*). Error bars indicate s.e.m. **c**,** d** Representative immunofluorescence images (**c**) and quantification (**d**) for MAG (top) and Fluoromyelin (bottom) in the striatum of 6-month-old *Gba1*^*f/f*^ and *Gba1*^*f/f*^*::cre* mice. Nuclei were stained by Hoechst (**, *p* < 0.01; *, *p* < 0.05 unpaired two-tailed Student’s t test; *n* = 6 randomly selected fields from 2 mice per genotype). Error bars indicate s.e.m. Scale bar = 50 μm. **e** Representative electron microscopy images of striatal and optic nerve axons from 6-month-old *Gba1*^*f/f*^ and *Gba1*^*f/f*^*::cre* mice. Scale bar = 1 μm. **f**,** g** G-ratio distribution of striatal and optic nerve myelinated axons (left panels), quantification of average g-ratio of myelinated axons (middle panels) and g-ratio distribution across axons divided into classes based on their diameter (right panels) (*, *p* < 0.05; **, *p* < 0.01; unpaired two-tailed Student’s t-test; striatum, n = 962 axon for *Gba1*^*f/f*^ from 3 mice and *n* = 641 axons for *Gba1*^*f/f*^*::cre* from 3 mice; optic nerves, n = 459 axon for *Gba1*^*f/f*^ from 3 mice and n = 624 axons for *Gba1*^*f/f*^*::cre* from 3 mice). Error bars indicate s.e.m. **h**,** i** Representative immunofluorescence for SOX10 in corpus callosum (CC) and striatum of 6-month-old *Gba1*^*f/f*^ and *Gba1*^*f/f*^*::cre* mice (**h**) and relative quantification (**i**). Scale bar = 50 μm (*n* = 4 mice per group). Error bars indicate s.e.m. **j**,** k** Representative immunofluorescence images for MBP on differentiated primary oligodendrocytes (**j**) and relative quantification of MBP^+^ covered area (**k**). Scale bar = 50 μm (*, *p* < 0.05; unpaired two-tailed Student’s t-test; *n* = 3 oligodendrocyte cultures derived from a single pup). Error bars indicate s.e.m. **l**,** m** Representative immunofluorescence image for MAG on differentiated primary oligodendrocytes (**l**) and relative quantification of the percentage of mature MAG^+^ oligodendrocytes on total MAG^+^ cells, based on their ramified morphology (**m**). Scale bar = 100 μm. (*, *p* < 0.05; unpaired two-tailed Student’s t-test; *n* = 4 oligodendrocyte cultures derived from a single pup). Error bars indicate s.e.m

MAG, MBP and PLP1 protein levels were reduced in total brain extracts of *Gba1*^*f/f*^*::cre* animals (Fig. 4a, b), paralleled by a significantly lower immunoreactivity for MAG protein at the level of myelinated axon bundles of the striatum, as well as of cerebellar WM tracts (Fig. 4c, d; Supplementary Fig. 3a, b). Not only the production of myelin-related proteins appeared to be affected by β-glucocerebrosidase dysfunction, but also compact myelin-related lipids, as indicated by fluoromyelin staining (Fig. 4c, d). Transmission electron microscopy (TEM) analysis of myelinated fibres sampled from different CNS regions, including striatum, optic nerve and cerebellum, denoted thinner myelin sheaths (Fig. 4e; Supplementary Fig. 3c). Altered myelination was assessed by morphometric analysis of g-ratio, a measure of myelin thickness based on the ratio between the axon diameter and the whole nerve fiber diameter (including myelin). Demyelination was confirmed by increased mean g-ratio in the striatal region of *Gba1*^*f/f*^*::cre* compared to *Gba1*^*f/f*^ mice (Fig. 4f). Interestingly, not only striatum but also optic nerve and cerebellum large caliber axons displayed significantly higher g-ratios (Fig. 4g; Supplementary Fig. 3d-f), mirroring a reduction in myelin thickness. Altered myelination was not accompanied by a depletion of oligodendrocytes as indicated by immunostaining for the transcription factor SOX10 (Fig. 4h). No significant differences in SOX10-positive cell density were detectable either in the striatum or corpus callosum (Fig. 4i). Supportive analysis on primary oligodendrocyte cultures derived from *Gba1*^*f/f*^ and *Gba1*^*f/f*^*::cre* littermate pups showed reduced MBP coverage upon β-glucocerebrosidase ablation in differentiating conditions (Fig. 4j, k), as well as reduced number of MAG-positive mature oligodendrocytes (Fig. 4l, m), further demonstrating impaired myelin formation upon genetic β-glucocerebrosidase loss-of-function. Western blot analysis showed a trend in MBP reduction while other myelin-related proteins, such as MAG and PLP1 appeared less affected (Supplementary Fig. 4a, b). Interestingly, both RT-qPCR and western blot analyses indicated that also early differentiation markers (Supplementary Fig. 4c), including *Pdgfra,* *Cspg4* and *Olig2* were affected in *Gba1*^*f/f*^*::cre* cultures when compared to controls (Supplementary Fig. 4a, b, d).

### Ablation of β-glucocerebrosidase in oligodendrocytes is sufficient to induce early markers of neurodegeneration

**Fig. 5:**
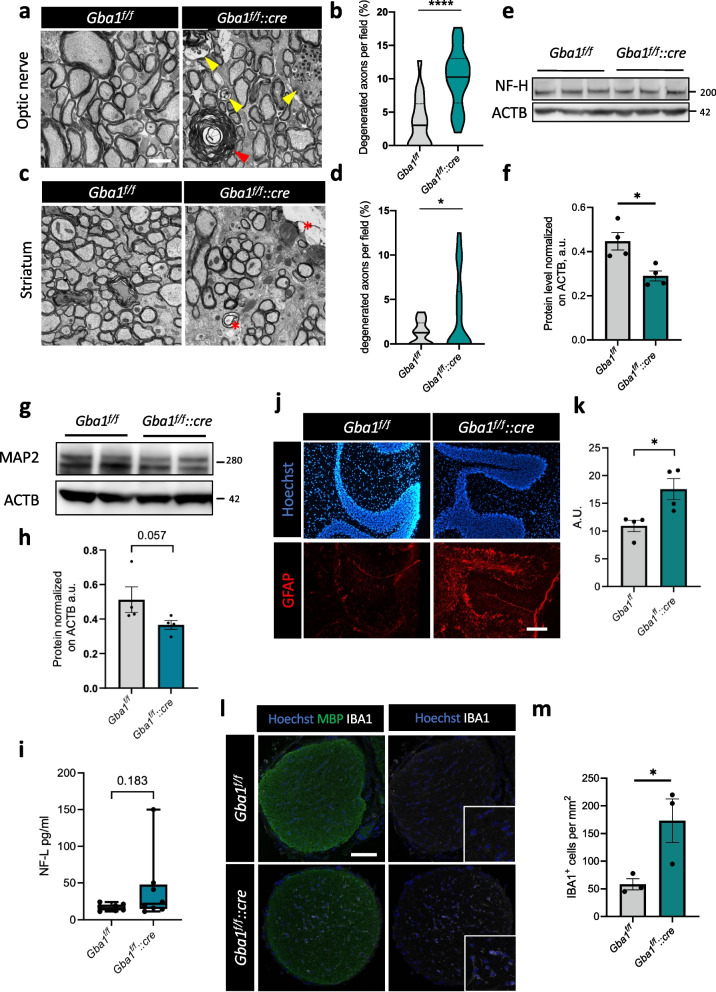
Lack of oligodendroglial β-glucocerebrosidase is sufficient to induce neurodegeneration, astrogliosis and neuroinflammation in vivo, **a, b** Representative electron microscopy images from optic nerve sections of 6-month-old *Gba1*^*f/f*^ and *Gba1*^*f/f*^*::cre* mice (**a**) and relative quantification of degenerated axons (**b**). Red arrowheads indicate myelin whorls; yellow arrowheads indicate electron-dense accumulated organelles (****, *p* < 0.0001; unpaired two-tailed Student’s t-test; *n* = 18 randomly selected fields from 3 mice for *Gba1*^*f/f*^ and *n* = 24 *Gba1*^*f/f*^*::cre* from 3 mice). Scale bar = 2 μm. **c**,** d** Representative electron microscopy images from striatum sections of 6-month-old *Gba1*^*f/f*^ and *Gba1*^*f/f*^*::cre* mice (**c**) and relative quantification of degenerated axons (**d**). Red asterisks indicate empty axons (*, *p* < 0.05; unpaired two-tailed Student’s t-test; *n* = 26 randomly selected fields from 3 mice for *Gba1*^*f/f*^ and *n* = 56 *Gba1*^*f/f*^*::cre* from 3 mice). **e**,** f** Representative western blot (**e**) for neurofilament H (NF-H) and relative densitometric quantification (**f**) of total brain protein extracts from 6-month-old *Gba1*^*f/f*^ and *Gba1*^*f/f*^*::cre* mice. β-actin (ACTB) was used as loading control (*, *p* < 0.05; unpaired two-tailed Mann–Whitney test; *n* = 4 mice). Error bars indicate s.e.m. **g**,** h** Representative western blot (**g**) for MAP2 and relative densitometric quantification (**h**) of total brain protein extracts from 6-month-old *Gba1*^*f/f*^ and *Gba1*^*f/f*^*::cre* mice. β-actin (ACTB) was used as loading control (unpaired two-tailed Mann–Whitney test; *n* = 4 mice). Error bars indicate s.e.m. **i** Absolute levels of serum neurofilament light chain (NF-L) in 6-month-old mice (unpaired Welch’s test, *n* = 8 mice). **j, k** Representative immunofluorescence (**j**) and relative quantification (**k**) for GFAP in cerebellum sections from 6-month-old *Gba1*^*f/f*^ and *Gba1*^*f/f*^*::cre* mice (*, *p* < 0.05; unpaired two-tailed Student’s t-test; *n* = 4 mice). Error bars indicate s.e.m. Scale bar = 100 μm. **l**,** m** Representative immunofluorescence for MBP and IBA1 in optic nerve sections from 6-month-old *Gba1*^*f/f*^ and *Gba1*^*f/f*^*::cre* mice (**l**) and relative quantification of IBA1^+^ microglial cell density (**m**). Scale bar = 100 μm (*, *p* < 0.05; unpaired two-tailed Student’s t-test; *n* = 3 mice). Error bars indicate s.e.m

Considering the altered myelination displayed by adult *Gba1*^*f/f*^*::cre* mice, we wondered whether the oligodendrocyte-axon unit might in turn be affected. TEM analysis of optic nerves highlighted the presence of several degenerating axons in *Gba1*^*f/f*^*::cre* mice, displaying features that include myelin whorls and dilated axons filled with electron-dense material or even empty ones (Fig. 5a, b; Supplementary Fig. 5a). Such alterations were detectable also at the level of the striatum of *Gba1*^*f/f*^*::cre* mice (Fig. 5c, d). Moreover, neurofilament heavy chain (NF-H) and microtubule-associated protein 2 (MAP2) levels were diminished in total brain extracts of *Gba1*^*f/f*^*::cre* mice, compared to control ones (Fig. 5e-h), pointing to neurodegeneration. Additionally, serum levels of NF-L chain used as a non-invasive method to detect neurodegeneration [54], showed a trend towards an increase in *Gba1*^*f/f*^*::cre* mice, compared to controls (Fig. 5i). In agreement with the presence of a degenerative insult going beyond the sole oligodendrocyte involvement, astrogliosis was detectable in *Gba1*^*f/f*^*::cre* cerebellum (Fig. 5j, k), together with an increase of inflammatory Iba1-positive cells in optic nerves (Fig. 5l, m), as well as in striatum, although at milder level (Supplementary Fig. 5b).

### Whole brain lipid dyshomeostasis is induced by loss of β-glucocerebrosidase in oligodendrocytes

**Fig. 6:**
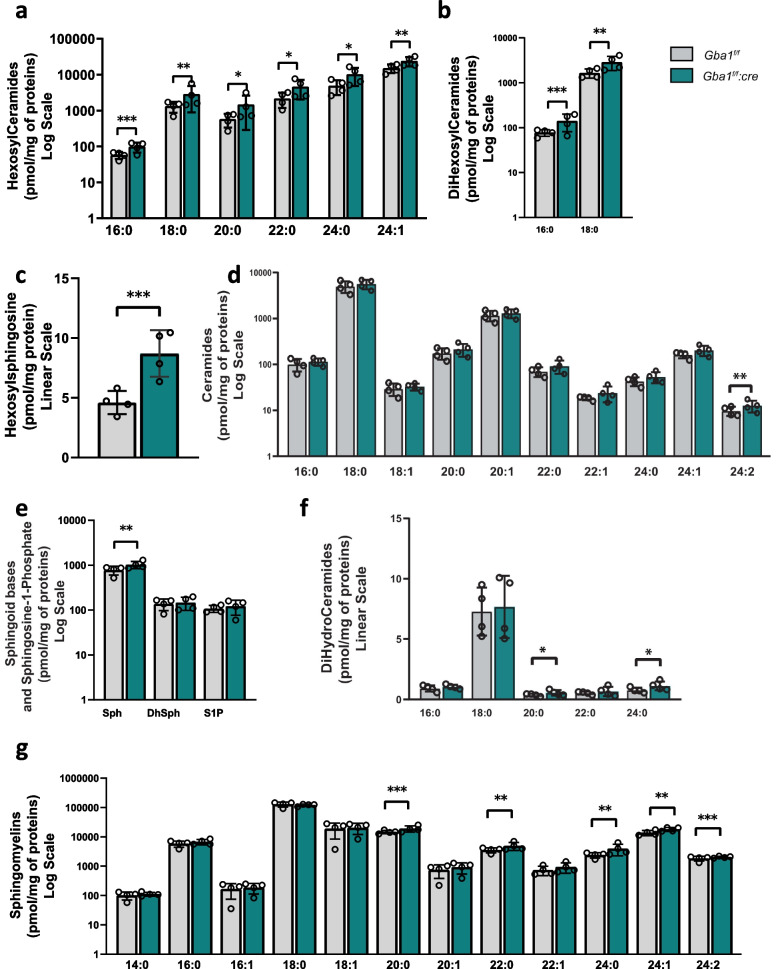
Global alterations in brain lipidome are displayed by mice lacking β-glucocerebrosidase in oligodendrocytes. **a-f** LC–MS sphingolipid levels in brain extracts from 6-month-old *Gba1*^*f/f*^ and *Gba1*^*f/f*^*::cre* mice. Mono- (**a**) and di-hexosylceramides (**b**), hexosylsphingosine (**c**) ceramides (**d**), sphingoid bases (Sph, sphingosine; DhSph, dihydrosphingosine) and sphingosine-1-phosphate (S1P) (**e**), dihydroceramides (**f**), sphingomyelins (**g**) were quantified based on ratio with internal standards as described in Methods (*, *p* < 0.05, **, *p* < 0.01, ***, *p* < 0.001; unpaired two-tailed Student’s t-test; *n* = 4). Error bars indicate s.e.m

Considering the major contribution of myelin to the total brain lipid content, we further investigated the presence of specific lipid moieties enrichment, caused by oligodendrocyte-specific β-glucocerebrosidase deficiency. A clear lipid dysregulation was detectable in *Gba1*^*f/f*^*::cre* brains, which displayed altered sphingolipid levels compared to controls. In particular, increased levels of hexosylceramides (HexCers) C16:0, C18:0, C20:0, C22:0, C24:0, C24:1 (Fig. 6a), dihexosylceramides (diHexCers) C16:0 and C18:0 (Fig. 6b) and HexSph (Fig. 6c) were detected. Among ceramides (Cer), only C24:2 increased (Fig. 6d). Sphingosine also resulted increased in *Gba1*^*f/f*^*::cre* brains (Fig. 6e), similarly to dihydroceramides (dhCers) C20:0, C24:0 (Fig. 6f) and very long chain sphingomyelins (SMs) (C20:0, C22:0, C24:0, C24:1, C24:2) (Fig. 6g). Immunoblot analysis for enzymes involved in the biosynthetic pathways of sphingolipids, such as serine palmitoyltransferase, long chain based subunit1 (SPTLC1), sphingomyelin synthase 1 (SMS1), acid and neutral sphingomyelinase (aSMase, nSMase), were performed, showing a trend toward decreased levels of aSMase and nSMase (Supplementary Fig. 6a), sustaining the increase of SMs. Interestingly, the expression of GlcCer synthase (*Ugcg*) was not altered in *Gba1*^*f/f*^*::cre* brains (Supplementary Fig. 6b), excluding the involvement of the biosynthetic pathway in lipid accumulation, rather pointing to a catabolic impairment. These results highlight the presence of brain lipidomic profile alterations with dysregulated levels of several species and increased level of unsaturated fatty acid chains, suggesting that a specific β-glucocerebrosidase ablation in oligodendrocytes impacts on brain lipid metabolism.

### α-synuclein oligomers accumulate in *Gba1*^*f/f*^*::cre* brains

**Fig. 7:**
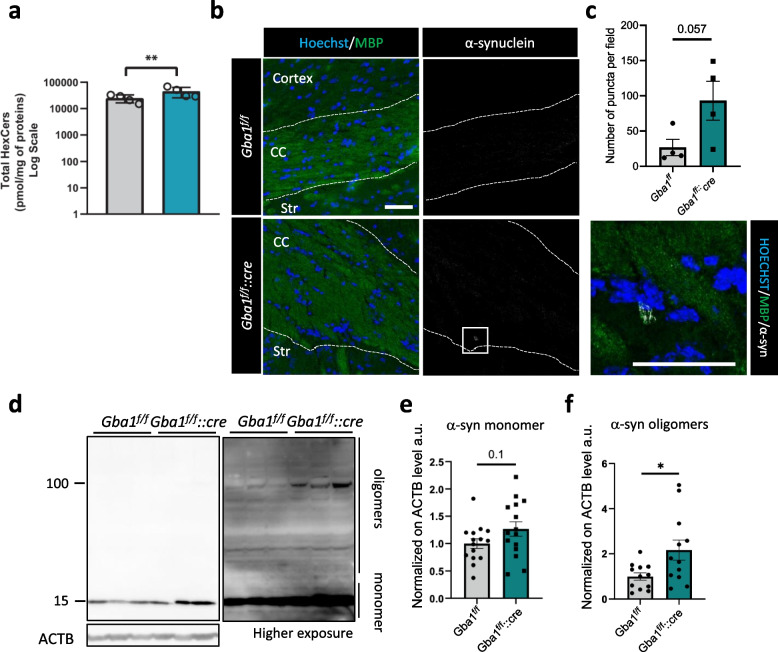
Altered lipid homeostasis in oligodendrocytes drives α-synuclein aggregation in vivo. **a** Levels of total hexosylceramide (HexCer) species in whole brain homogenates from 6-month-old *Gba1*^*f/f*^ and *Gba1*^*f/f*^*::cre* mice (**, *p* < 0.01; unpaired two-tailed Student’s t-test; *n* = 4). Error bars indicate s.d. **b**,** c** Representative immunofluorescence images for Triton X-100-insoluble α-synuclein and MBP in the corpus callosum from 6-month-old *Gba1*^*f/f*^ and *Gba1*^*f/f*^*::cre* mice (***b***) and relative quantification of the number of puncta detected per field (Unpaired two-tailed Mann–Whitney test; *n* = 4 mice). Error bars indicate s.e.m. CC, corpus callosum, Str, striatum. Scale bar = 50 μm. **d-f** Representative western blot of α-synuclein monomer and oligomers (**e**) in total brain protein extracts from 6-month-old *Gba1*^*f/f*^ and *Gba1*^*f/f*^*::cre* mice and relative densitometric quantification (**e**, **f**). β-actin (ACTB) was used as loading control. Lower and higher exposure are shown to highlight the presence of oligomers as well as of the monomer (*, *p* < 0.05; unpaired two-tailed Student’s t-test; *n* = 15 mice for α-synuclein monomers and *n* = 12 for α-synuclein oligomers). Error bars indicate s.e.m

β-glucocerebrosidase deficiency was previously widely associated with α-synuclein accumulation, becoming an acknowledged hallmark of neurodegeneration in synucleinopathies [16–18]. Since increased levels of GlcCer were shown to elicit α-synuclein aggregation in GD [19], the accumulation of HexCers (GlcCer and GalCer) observed in *Gba1*^*f/f*^*::cre* brains compared to controls (Fig. 7a), prompted us to investigate α-synuclein. Accordingly, we detected an increased number of α-synuclein-positive puncta in the corpus callosum of *Gba1*^*f/f*^*::cre* mice, compared to controls (Fig. 7b, c). Furthermore, western blot analysis of *Gba1*^*f/f*^*::cre* total brain extracts revealed increased levels of α-synuclein oligomers, rather than monomers (Fig. 7d-f).

### Oligodendrocyte-specific β-glucocerebrosidase dysfunction causes mild functional alterations in vivo

**Fig. 8:**
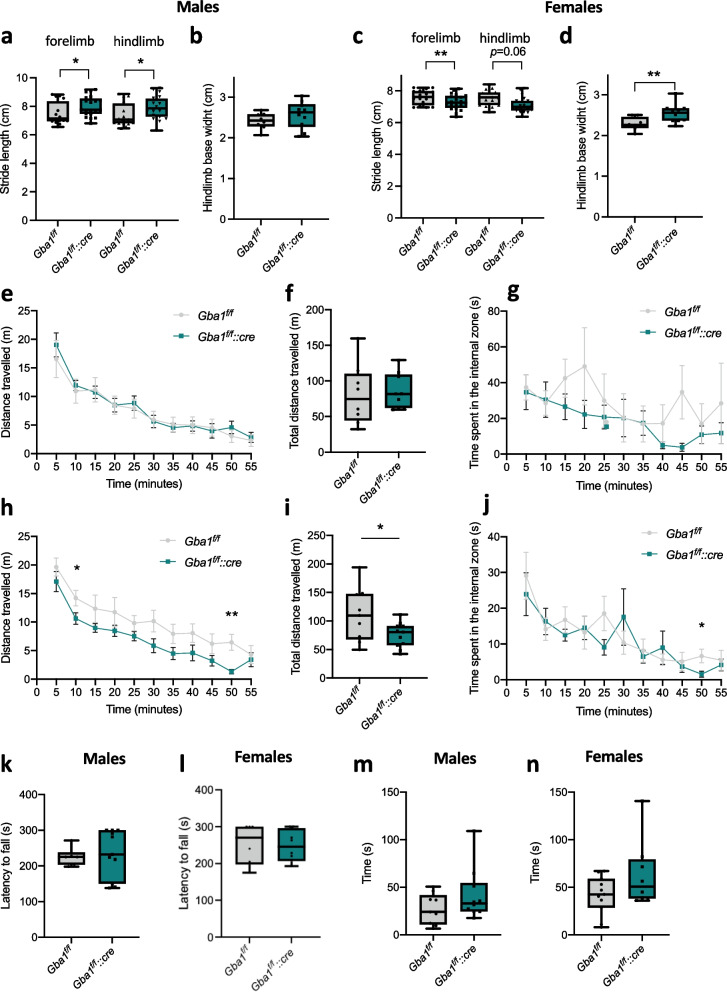
Behavioral analysis of *Gba1*^*f/f*^*::cre* shows altered locomotor activity and worse olfactory performance. **a**,** b** Analysis of gait in 6-month-old *Gba1*^*f/f*^ and *Gba1*^*f/f*^*::cre* male mice. Stride length (**a**) and hindlimb base width (**b**) were measured (*, *p* < 0.05; unpaired two-tailed Student’s t-test; *n* = 16 *Gba1*^*f/f*^ and *n* = 20 *Gba1*^*f/f*^*::cre* mice). Error bars indicate maximum and minimum values.** c**,** d** Analysis of gait in 6-month-old *Gba1*^*f/f*^ and *Gba1*^*f/f*^*::cre* female mice. Stride length (**c**) and hindlimb base width (**d**) were measured (**, *p* < 0.01; Unpaired two-tailed Student’s t-test; n = 16 *Gba1*^*f/f*^ and n = 20 *Gba1*^*f/f*^*::cre* mice). Error bars indicate maximum and minimum values.** e–g** Open field locomotion test in 6-month-old *Gba1*^*f/f*^ and *Gba1*^*f/f*^*::cre* male mice. The total distance traveled (**e, f**) and time spent in the internal zone (**g**) were measured (*n* = 8 *Gba1*^*f/f*^ and n = 9 *Gba1*^*f/f*^*::cre* mice). **h-j** Open field locomotion test in 6-month-old *Gba1*^*f/f*^ and *Gba1*^*f/f*^*::cre* female mice. The total distance traveled (**h**, **i**) and time spent in the internal zone (**j**) were measured (*, *p* < 0.05; **, *p* < 0.01; unpaired two-tailed Student’s t-test; *n* = 9 *Gba1*^*f/f*^ and *n* = 11 *Gba1*^*f/f*^*::cre* mice). Error bars indicate maximum and minimum values.** k, l** Rotarod performance of 6-month-old *Gba1*^*f/f*^ and *Gba1*^*f/f*^*::cre* male (**k**) and female (**l**) mice. Results are shown as the average of the best performance from each (unpaired two-tailed Student’s t-test; *n* = 6 *Gba1*^*f/f*^ and *n* = 10 *Gba1*^*f/f*^*::cre* male mice and *n* = 6 *Gba1*^*f/f*^ and *n* = 11 *Gba1*^*f/f*^*::cre* female mice). Error bars indicate maximum and minimum values. **m**,** n** Performance in the buried pellet test for olfactory testing of male and female mice at 6 months of age is shown (unpaired two-tailed Student’s t-test; *n* = 11 *Gba1*^*f/f*^ and n = 8 *Gba1*^*f/f*^*::cre* mice for females and *n = *9 *Gba1*^*f/f*^ and *n* = 10 *Gba1*^*f/f*^*::cre* mice). Error bars indicate maximum and minimum values

The occurrence of oligodendrocyte/axonal unit alterations often leads to detectable functional impairment [55]. Therefore, we tested at first motor behaviour, one of the most affected outcomes upon neurodegeneration onset [56]. Gait analysis highlighted longer stride lengths in *Gba1*^*f/f*^*::cre* male mice, compared to controls (Fig. 8a), while alterations of the base width were less evident (Fig. 8b). Conversely, female *Gba1*^*f/f*^*::cre* mice displayed shorter strides when compared to controls (Fig. 8c), along with a larger base width (Fig. 8d). Open field locomotor activity tests did not show any major alteration in male mice in terms of general locomotor activity during the time of recording (Fig. 8e), nor in the total distance travelled (Fig. 8f). No major differences were detected in the time spent in the internal or external zone suggesting no effects in anxiety-like phenotypes (Fig. 8g; Supplementary Fig. 7a). In contrast, a lower locomotor activity in *Gba1*^*f/f*^*::cre* compared to controls was observed in female mice (Fig. 8h, i). A slight increase in the time spent in the internal zone was also detectable in the last minutes of recording, in females (Fig. 8j, Supplementary Fig. 7b). Motor coordination was not affected in either male or female *Gba1*^*f/f*^*::cre* mice compared to control ones, as revealed by rotarod test (Fig. 8k, l; Supplementary Fig. 7c, d). Since hyposmia was previously reported as a prodromic feature in GBA1-related Parkinson’s disease [57], we also measured the latency to find a buried food pellet. This test showed an interesting, although not significant, trend for both *Gba1*^*f/f*^*::cre* male and female mice spending more time to find the pellet compared to controls (Fig. 8m, n), thus pointing to a mildly reduced olfactory function. Overall, these data demonstrate sex-dichotomous effect of GBA1 deletion especially in locomotor activity parameters.

## Discussion

Β-glucocerebrosidase is a lysosomal enzyme that catalyses the hydrolysis of GlcCer into its two components, glucose and ceramide. In humans, mutations affecting β-glucocerebrosidase catalytic activity are known to cause GD, a rare LSD with neurological involvement and encompassing parkinsonism-like symptoms [10]. Heterozygous mutations are known as the most prominent genetic risk factor for a form of early onset PD, that also has a higher prevalence in GD patients compared to the general population [58]. The mechanism through which GBA1 mutations drive the neuronopathic phenotype that reproduces PD neurodegenerative hallmarks is multi-faceted and not entirely understood [20]. So far, PD has been mostly considered a grey matter disease, but increasing evidence supports the involvement of different cell types in the aetiology of this synucleinopathy. With specific reference to the *GBA1* mutations in PD, experimental evidence highlighted an impact on astrocytes, characterised by altered degradative capacity and defective inflammatory response [24, 25] and on microglia, whose ability to protect neurons from oxidative stress is defective upon β-glucocerebrosidase inactivation [26].

In this work we report for the first time a role for β-glucocerebrosidase in oligodendrocytes. Our results show that the loss of lysosomal glucocerebrosidase function specifically in myelinating glia impairs cell differentiation in vitro and the proper maintenance of myelin sheaths in vivo. In vitro experiments helped elucidate cellular processes affected by β-glucocerebrosidase dysfunction in myelinating glia and how this could impact on cell differentiation. Chemical inhibition of β-glucocerebrosidase in the Oli-neu cell line affected the autophagic-lysosomal pathway, as reported for other cell types [16, 48, 59]. Indeed, CBE treatment in oligodendrocytes induced a decrease in the number of acidified lysosomes together with defective degradation of autophagic cargoes, as shown by the increased co-localization of p62 and LAMP1 in enlarged structures. β-glucocerebrosidase inhibition in Oli-neu also induced GlcCer and α-synuclein accumulation as well as lipid dyshomeostasis detectable as an altered cholesterol distribution. Moreover, CBE treatment turned into reduced oligodendrocyte maturation, in keeping with a decreased number of mature oligodendrocytes in primary cultures from *Gba1*^*f/f*^*::cre* pups, paralleled by an increased g-ratio in adult GBA1 mutant brains, supporting similar effects exerted by chemical inhibition and genetic ablation of the enzyme. Indeed, myelin synthesis widely relies on a functional and responsive lysosomal compartment, as myelin-related proteins, such as PLP, MAG and MOG, are targeted or recycled to the plasma membrane through the late endosome/lysosomal or endosomal pathway [60, 61]. Moreover, induction of the autophagy/lysosomal process is known to promote oligodendrocyte maturation [62] and lysosomes are increasingly recognized as central drivers in lipid trafficking and catabolism for the production and recycling of myelin [63, 64]. On the other hand, lysosomal alkalinization was recently demonstrated to be sufficient to induce a delay in oligodendrocyte maturation [65], supporting a direct impact of the lysosomal impairment generated by β-glucocerebrosidase dysfunction in this context. Interestingly, disrupted cholesterol trafficking, leading to a punctate distribution as displayed by Oli-neu cells upon β-glucocerebrosidase inactivation, was also found to reduce oligodendrocyte differentiation in an Alzheimer’s disease in vitro context, a defect that can be recovered by cyclodextrin that facilitates cholesterol transport to the membrane [66].

Our data also show that treatment with CBE increases intracellular α-synuclein levels in Oli-neu cells. Although primarily studied in neurons, α-synuclein is also expressed by oligodendrocyte progenitor cells (OPCs) and oligodendrocytes [67–69], and a reduced lysosomal degradation of toxic intracellular components in synucleinopathies can result in the formation of protease-resistant α-synuclein aggregates, hallmarks of both PD and MSA [70, 71]. Of note, α-synuclein expression was shown to negatively influence primary oligodendrocyte maturation [72].

Despite β-glucocerebrosidase inhibition in Oli-neu cells hampered their differentiation, it did not increase apoptotic events, similarly to what observed in our in vivo mouse model displaying reduced myelination, but not a decreased number of SOX10^+^ oligodendrocytes in the striatum and corpus callosum. This is in keeping with observations in post-mortem samples from subjects with MSA, an α-synucleinopathy characterised by oligodendroglial α-synuclein inclusions, in which oligodendrocyte maturation is impaired without affecting cell viability [72].

Not only β-glucocerebrosidase dysfunction in oligodendrocytes alters their homeostasis, but our in vivo studies show that it also triggers neurodegeneration, highlighting a non-cell autonomous impact, affecting neurons and other glial cell types. Axonal degeneration in optic nerves and striatal regions were detected in our model at six months of age, supporting an early contribution of myelinating glia to β-glucocerebrosidase systemic disorders and thus suggesting the involvement of multiple cell types in the onset of neurodegeneration. Alterations in the WM of GD patients were indeed associated with cognitive impairment [73], in line with reports suggesting that in PD patients with cognitive impairment, MRI-detectable WM structural alterations precede grey matter degeneration [74–76].

Primary oligodendrocyte alterations are known to impact on neuronal homeostasis and survival with a range of influences, including impaired axonal transport by disrupted contacts between axons and oligodendroglia [77] or loss of metabolic supply [78, 79]. Moreover, perturbations of oligodendrocyte functions might induce brain lipid dyshomeostasis and inflammation, leading to the development and progression of neurodegeneration [80].

The lack of β-glucocerebrosidase is expected to result in unbalanced lipid metabolism due to the accumulation of its substrates GlcCer, GlcSph and glucosylcholesterol [59]. In our models, an altered lipid profile was detectable in CBE-treated Oli-neu cells, and was displayed at a greater extent in brain tissues from *Gba1*^*f/f*^*::cre* mice. Among the three mentioned substrates, GlcSph appeared the most affected by β-glucocerebrosidase dysfunction, contributing to the increased levels of detectable HexSph species in cells and in brains. Indeed, this is in line with elevated GlcSph levels detected in PD patients bearing *GBA1* mutations or in the related murine models. While an accumulation of GlcCer was not consistently reported, GlcSph increase resulted significant and more reliable in different conditions, although depending on patients’ age and brain regions [81–83]. The lipidomic analysis of *Gba1*^*f/f*^*::cre* brains showed that, at a relatively early stage in life, several lipid species such as mono- and di-hexosylceramides (GlcCer, GalCer and LacCer) as well as Cers, dhCers, sphingosine and SMs, display a strong imbalance. According to our data, most of the lipid moieties found increased in *Gba1*^*f/f*^*::cre* brains are expected to arise from a decrease in their lysosomal-related catabolic pathways in oligodendrocytes rather than from an enhanced synthesis. Indeed, a lysosomal impairment determining a loss of acidification, as seen in Oli-neu cells upon β-glucocerebrosidase inactivation, is at the base of lysosomal storage disorders affecting WM, such as Krabbe disease, lacking functional galactocerebrosidase [84] and Niemann-Pick type A and B, lacking acid sphingomyelinase [85].

The altered lipid profiles might contribute to the neurodegenerative features displayed by *Gba1*^*f/f*^*::cre* mice, as it is well recognized that the buildup of bioactive lipids exerts a plethora of toxic stimuli. Interestingly, increased levels of dhCer led to neurodegeneration in a *Drosophila* model lacking the enzyme dihydroceramide desaturase (human DEGS1, *Drosophila* Ifc) representing the final step of ceramide de novo synthesis [86] and *DEGS1* mutations were associated with hypomyelination and leukodystrophy in humans [87]. Moreover, the complex and intertwined fate of glial cells and neurons in the CNS could also account for crosstalks, in which an excess of GlcCer in oligodendrocytes might activate microglia, through specific receptors with a mechanism recently reported to drive phagocytosis of neurons and axonal degeneration [88]. Furthermore, GlcCer can stabilize α-synuclein toxic multimers by mimicking their structure and acting as a scaffold [89, 90]. Additionally, the increase of ceramide species can generate neuronal and oligodendrocyte damage [91, 92] and this was also considered to play a role in PD pathophysiology, as altered ceramide levels were found in post-mortem samples of PD patients’ brain and serum [93, 94].

The molecular hallmarks of neurodegeneration in *Gba1*^*f/f*^*::cre* mice are accompanied by functional alterations that well fit with GBA1-related progressive phenotypes. In the early stages of PD, patients may exhibit hyposmia, sleep disturbance and autonomic dysfunction [95]. These symptoms can then be followed by characteristic motor dysfunctions such as rigidity, tremor and gait abnormalities [96]. Accordingly, our mutant mice displayed altered stride lengths and higher base width compared to controls, paralleled by a reduced total distance travelled particularly in females. Altered stride length and wider base width are often reported in mouse models of PD [97, 98]. While not all the *Gba1*-related PD mouse models mirror the human gait deficits [99], it has been demonstrated that pharmacological dopamine depletion or dopamine receptor inhibition in the striatal region, thereby targeting the most affected circuitries in PD mouse model, impacted on gait by reducing stride length [100]. As well, it has been also demonstrated that an increase in α-synuclein expression was able to impact on gait by reducing stride length and the distance travelled in open field arena [101].

Prodromal symptoms of sleep disorder and hyposmia can be triggered in mice by overexpressing a mutant aggregation-prone α-synuclein [102]. In our mice lacking β-glucocerebrosidase in oligodendrocytes we were able to detect slight signs of hyposmia at six months of age together with gait alterations, in parallel with an increased number of degenerating neurons and accumulation of α-synuclein oligomers in the brain from *Gba1*^*f/f*^*::cre* mice. Since murine models of genetic forms of PD can require longer time to develop olfactory dysfunction, compared to chemically-induced ones [103], and considering that *Gba1* deletion in our model is restricted to oligodendrocytes, it is conceivable that older *Gba1*^*f/f*^*::cre* mice could display a much aberrant olfactory phenotype. Interestingly, behavioural tests highlighted sex-related differences. In this context, while the risk of developing idiopathic PD is doubled in men compared to women, it was also reported that clinical symptoms can differ, since clinical outcomes and progression appears to be more severe in women than in men [104]. By the way, a gender effect in GBA1-related PD has not been established with certainty yet [105, 106] therefore it is difficult to ascribe the observed differences to a specific cause. However, differences were recently described in microglial response to CBE treatment [107], suggesting that also other cells can account for such specificity.

## Conclusions

Altogether, our study highlights for the first time a role of oligodendrocytes in the onset and development of GBA1-related neurodegenerative features. Lysosomal dysfunction in oligodendrocytes leads to impaired maturation and to toxic substrate accumulation, including lipids and α-synuclein aggregates, ultimately triggering a neurodegeneration that is detectable through histological, molecular and mild functional hallmarks. Our study sheds light on the contribution of oligodendrocytes in CNS diseases and supports the need for the characterization of all the actors playing a role in neurodegenerative diseases, pointing at oligodendrocytes as novel key cellular targets for therapeutic purposes. 

### Supplementary Information

**Supplementary Material 1.**

## Data Availability

All data generated or analysed during this study are included in this published article [and its supplementary information files].

## Abbreviations

aSMase: Acid sphingomyelinase
CBE: Conduritol B epoxide
Cer: Ceramide
CNS: Central nervous system
Cre: Cre-recombinase
dbcAMP: Dibutyryl-cAMP
dhCer: Dihydroceramide
diHexCers: Dihexosylceramides
Gb3: Globotriaosylceramide
GBA1: β-Glucocerebrosidase
GD: Gaucher disease
GlcCer: Glucosylceramide
HexCers: Hexosylceramides
GlcSph: Glucosylsphingosine
HexSph: Hexosylsphingosine
HexChol: Hexosylcholesterol
IDS: Iduronate 2-sulfatase
LacCer: Lactosylceramide
LAMP1: Lysosomal-associated membrane protein-1
LSDs: Lysosomal storage diseases
nSMase: Neutral sphingomyelinase
PD: Parkinson’s disease
SM: Sphingomyelin
SMS1: Sphingomyelin synthase 1
SPTLC1: Serine palmitoyltransferase, long chain based subunit 1
WM: White matter
