# Supplementary material for: GBA1 inactivation in oligodendrocytes affects myelination and induces neurodegenerative hallmarks and lipid dyshomeostasis in mice
Source: Mol Neurodegener. 2024 Mar 7;19:22. doi: 10.1186/s13024-024-00713-z (PMC10921719; doi:10.1186/s13024-024-00713-z)

Supplementary Material

Supplementary Tables

Supplementary Table 1. Primers used for genotyping

Target	Primer Forward 5' → 3'	Primer Reverse 5' → 3'	Amplicon Size
Cnp1 wild type allele	CATAGCCTGAAGAACGAGA	CCCAGCCCTTTTATTACCAC	400 bp
Cnp1-cre allele	GCCTTCAAACTGTCCATCTC	CCCAGCCCTTTTATTACCAC	700 bp
$Gba1^{f/f}$ or $Gba1^{\Delta ex9\text{-}11}$	GTACGTTCATGGCATTGCTG TTCACT	ATTCCAGCTGTCCCTCGTCT CC	2100 bp ($Gba1^{f/f}$) or 700 bp (*Δex9-11*)

Supplementary Table 2. List of antibodies used for immunofluorescence (IF) and/or western blotting.

Protein	Application	Species	Supplier	Catalogue No.
MAG	WB, IF	Rabbit	Cell Signaling	9043
PLP	WB, IF	Rabbit	Novus Biologicals	NBP1-87781
CNPase	WB, IF	Rabbit	Cell Signaling	5664
GBA1	WB	Mouse	Santa Cruz	sc-166407
Cleaved caspase 3	WB	Rabbit	Cell Signaling	9661
LAMP1	WB, IF	Rat	DSHB	1D4B
β-actin	WB	Mouse	Sigma-Aldrich	A5316
Vinculin	WB	Mouse	Sigma-Aldrich	V4505
IDS	WB	Mouse	Novus Biologicals	NBP2-01761
TH	WB	Rabbit	Sigma-Aldrich	AB152
p62/SQSTM1	IF	Guinea Pig	Progen	GP62-C
Phospho-α-synuclein	WB, (IF)	Rabbit	Abcam	ab52153
α-synuclein	WB	Rabbit	Abcam	ab52168
MBP	WB, IF	Rat	Abcam	ab7349
Olig2	WB, (IF)	Rabbit	Millipore	AB9610
PDGFRα	WB	Rat	Invitrogen	14-1401-82
GlcCer	IF	Rabbit	Glycobiotech	RAS_001
GFAP	IF	Mouse	Millipore	MAB3402
IBA1	IF	Rabbit	FUJIFILM Wako	019-19741
MAP2	WB	Rabbit	GeneTex	GTX133109
Neurofilament H	WB	Rabbit	Millipore	AB1991

Supplementary Table 3. List of forward and reverse primers used for RT-qPCR.

Target	Primer Forward 5'→ 3'	Primer Reverse 5'→ 3'
Actb	CTAAGGCCAACCGTGAAAAG	ACCAGAGGCATACAGGGACA
Cnp	CTGGAGATGAACCCAAGGAGAAGC	CGATCTCTTCACCACCTCCTGCTCTG
Cspg4	CTCACACAGAGGAGCCCTGGA	AGACGGTCAACTTCCGGGTG
Gapdh	ATCCTGCACCACCAACTGCT	GGGCCATCCACAGTCTTGTG
Gba1 (catalytic coding region)	CTCTGCAGTTGTGGTCGTGT	GGAAGCCCAGGTCAGGATCA
Gba1	TCCTTGCTTTGTCCCCACCT	AGTCGTTAGGGGTGTCAGCA
Gba2	GATGGCTGTTCTGTGTGGGG	AATAGCGTCCGTTCCACAGC
Gpr17	TCTTCTGCAAGAGCCACAAGATGAA	GGGGGTCTCTTGTCCCGCATT
Mag	GCTCCTCATTGCAGTTCCCTGA	ATCCAAAACAGCGGCAGGGT
Mbp	GCTCCCTGCCCCAGAAGT	TGTCACAATGTTCTTGAAGAAATGG
Olig2	CCCCGAAAGGTGTGGATGCT	GGCCCCAGGGATGATCTAAGC
Pdgfra	CGGGTTTTGAGCCCATTACTGT	TAGCTCCTGAGACCTTCTCCTTC
Plp1	CACTTACAACTTCGCCGTCCT	CACTTACAACTTCGCCGTCCT
Rpl7l1	AGAGCAGGAGCAGGTTTTCC	CAGCCAATGAGGGAACTCGT
Ugcg	ACCGAGGTTGGAGGTTTTCAA	TTCTCAATTTGGTCCACCTGA

Supplementary Table 4. MRM transitions of hexosylsphingosine (HexSph), hexosylceramides (Hexcers) and hexosylcholesterol (HexChol)**.**

Compound	Transition (Q1>Q3)	Cone Voltage (V)	Collision Energy (eV)
LysoGb2 (I.S.)	624.3>282.2	22	40
HexSph	462.3>282.2	22	40
Glucosyl (β)ceramide (d18:1/12:0) (I.S.)	644.5>264.4	22	40
HexCer d18:1 C16:0	700.7>264,4	22	40
HexCer d18:0 C16:0	702.7>266.4	22	40
HexCer d18:1 C18:0	728.7>264.4	22	40
HexCer d18:10 C18:0	730.7>266.4	22	40
HexCer d18:1 C20:0	756.7>264.4	22	40
HexCer d18:1 C22:0	784.8>264.4	22	40
HexCer d18:1 C24:0	812.9>264.4	22	40
HexCer d18:0 C24:0	814.9>264.4	22	40
HexCer d18:1 C24:1	810.9>264.4	22	40
HexChol	566.5>369.3	50	16

Supplementary Table 5. MRM transitions of sphingosine, dihydrosphingosine, sphingosine-1-phosphate, dihydro-sphingosine-1-phosphate and hexosylcholesterol

Compound	Transition (Q1>Q3)	Cone Voltage (V)	Collision Energy (eV)
LysoGb2 (I.S.)	624.3>282.2	22	40
HexSph	462.3>282.2	22	40
HexChol	566.5>369.3	50	16
Sphingosine d17:1 (I.S.)	286.40>250.40	40	16
Sphingosine d18:1	300.40>264.40	40	16
Sphingosine d18:0	302.4>266.4	40	16
Sphingosine-1-phosphate d17:1 (I.S.)	366.40 > 250.40	20	16
Sphingosine-1-phosphate d18:1	380.40 > 264.40	20	16
Sphingosine-1-phosphate d18:0	382.4 > 266.4	20	16

Supplementary Figures

Supplementary Figure Legends

Suppl. Fig. 1 Analysis of the impact of CBE on gene expression of lysosomal enzymes and on lysosomal function in Oli-neu cells. (a) qRT-PCR analysis of *Galc* and *Ids* mRNA from Oli-neu cells treated with dbcAMP for three days. *Actb* was used as housekeeping gene (*, $p < 0.05$; Kruskal-Wallis test and Dunn's test for multiple comparison, n = 3 independent experiments). Error bars indicate s.e.m.. **(b)** MRM-MS quantitation of hexosylcholesterol in differentiated vehicle (veh) and CBE treated Oli-neu (n = 4 independent experiments). The whiskers of the box plot represent the minimum and the maximum data values. **(c)** Immunofluorescence staining on undifferentiated (t0) Oli-neu cells upon vehicle (veh) or CBE treatment. Scale bar = 100 µm. **(d)** Representative anti-LAMP1 staining (red) on differentiated Oli-neu cells performed after incubation with LysoTracker (green). Scale bar = 50 µm. Nuclei were stained with Hoechst. **(e, f)** representative western blot (e) and relative densitometric quantification (f) of LAMP1 protein levels in Oli-neu cells upon vehicle (veh) or CBE treatment at t3; β-actin (ACTB) was used as loading control (n = 4 independent experiments). Error bars indicate s.e.m.

Suppl. Fig. 2 Validation of oligodendrocyte- and Schwann cell-specific *Gba1* deletion by PCR, western blotting and enzymatic activity assay. (a) Schematic diagram of the strategy to generate the conditional deletion of exons 9–11 of the *Gba1* gene. The *Gba1*$^{f/f}$ mouse line (left), bearing LoxP sequences (yellow triangles) flanking the exons coding for the active site of the enzyme was crossed with the *Cnp1-cre* mouse line (right), expressing cre recombinase under the control of endogenous *Cnp1* promoter. Primers annealing on the exon 8 of *Gba1* and exon 8 of *Mtx1* (red arrows) generate a 2100 bp product in *Gba1*$^{f/f}$ mice, and a smaller product of 700 bp upon cre-mediated recombination. **(b)** PCR performed on genomic DNA from brain samples of 1 month-old *Gba1*$^{f/f}$ and *Gba1*$^{f/f}$*::cre* mice using the primers shown in (a). A standard DNA ladder was loaded in the last lane of the gel. **(c)** Representative western blot for GBA1 and relative densitometric quantification on total protein extracts from primary oligodendrocyte cultures derived from *Gba1*$^{f/f}$ and *Gba1*$^{f/f}$*::cre* pups (*, $p < 0.05$; unpaired two-tailed Student's t test; n = 14 *Gba1*$^{f/f}$ and n = 16 *Gba1*$^{f/f}$*::cre* independent primary oligodendrocyte cultures). Error bars indicate s.e.m. **(d)** Representative western blot for GBA1 and relative densitometric quantification on total protein extracts from primary Schwann cell cultures derived from *Gba1*$^{f/f}$ and *Gba1*$^{f/f}$*::cre* mice (n = 6 *Gba1*$^{f/f}$ and n = 2 *Gba1*$^{f/f}$*::cre* independent primary Schwann cell cultures). Error bars indicate s.e.m. N.D, not detectable. **(e, f)** Representative western

blot for GBA1 and relative densitometric quantification on total brain (e) and cerebellum (f) extracts from 6-month-old *Gba1$^{f/f}$* and *Gba1$^{f/f}$::cre* mice (*, $p < 0.05$; unpaired two-tailed Mann-Whitney U test; n = 4 samples per group in (e) and n = 6 samples per group in (f)). Error bars indicate s.e.m. **(g)** Enzymatic activity assay performed on protein extracts from central nervous system areas of *Gba1$^{f/f}$* and *Gba1$^{f/f}$::cre* (*, $p < 0.05$; ***, $p < 0.001$; unpaired two-tailed Student's t test; for total brain n = 4 *Gba1$^{f/f}$* and n = 7 *Gba1$^{f/f}$::cre*; for cerebellum n = 3 *Gba1$^{f/f}$* and n = 3 *Gba1$^{f/f}$::cre*). Error bars indicate s.e.m. Cereb., cerebellum; Sp. cord, spinal cord.

Suppl. Fig. 3 Quantification of MAG fluorescence and g-ratio of myelinated axons in the cerebellum of *Gba1$^{f/f}$* and *Gba1$^{f/f}$::cre* mice. (a, b) Representative immunofluorescence (a) and relative quantification (b) of MAG in the cerebellum of *Gba1$^{f/f}$* and *Gba1$^{f/f}$::cre* mice (*, $p < 0.05$; unpaired Student t test, n = 4 mice). Error bars indicate s.e.m. **(c)** Representative TEM images of cerebellum myelinated axons *Gba1$^{f/f}$* and *Gba1$^{f/f}$::cre* mice. Scale bar = 1 μm **(d-f)** Dot-plot of g-ratios of cerebellum myelinated axons (d), average g-ratio of myelinated axons (e) and g-ratio across axons divided into classes based on their diameter (f) (*, $p < 0.05$; unpaired two-tailed Student's t-test; n = 477 axons from 3 mice for *Gba1$^{f/f}$* and n = 323 axons from 3 mice for *Gba1$^{f/f}$::cre*). Error bars indicate s.e.m.

Suppl. Fig. 4 Western blot and qPCR analysis of lineage-specific markers in *Gba1$^{f/f}$* and *Gba1$^{f/f}$::cre* primary oligodendrocyte cultures. (a, b) Representative western blot (a) and relative densitometric quantification (b) on total protein extracts from differentiated primary oligodendrocytes. The levels of MAG, OLIG2, PLP1, MBP and PDGFRα were analysed. β-actin (ACTB) was used as loading control (unpaired two-tailed Student's t-test; n = 7-15 independent primary oligodendrocyte cultures). Error bars indicate s.e.m. **(c)** Schematic diagram showing the positivity for stage-specific markers along the oligodendrocyte differentiation trajectory. **(d)** Heat-map showing mRNA expression of oligodendrocyte stage-specific markers. Data is shown as fold change of *Gba1$^{f/f}$::cre* compared to *Gba1$^{f/f}$* derived primary oligodendrocytes. (*, $p < 0.05$; unpaired two-tailed Student's t-test; n = 6-9 independent primary oligodendrocyte cultures).

Suppl. Fig. 5 Ultrastructural analysis of *Gba1$^{f/f}$::cre* optic nerves showing axonal degeneration. (a) Representative transmission electron microscopy images of 6-month-old *Gba1$^{f/f}$::cre* optic nerves sections. Red asterisk indicates empty axons. Red arrowheads indicate myelin whorls. Yellow arrowheads show electron-dense accumulated organelles within axons. **(b)** Quantification of IBA1^{+}

cells detected by immunofluorescent staining of striatum from 6-month-old *Gba1*$^{f/f}$ and *Gba1*$^{f/f}$*::cre* mice (unpaired two-tailed Student's t-test; n = 5 mice).

Suppl. Fig. 6 Quantification of enzymes involved in ceramide metabolism. (a) Densitometric analysis of enzymes involved in ceramide metabolism in total brain protein extracts from 6-month-old *Gba1*$^{f/f}$ and *Gba1*$^{f/f}$*::cre* mice (unpaired two-tailed Student's t-test; n = 4 mice per genotype). **(b)** qRT-PCR for *Ugcg* in total brain RNA extracts from 6-month-old *Gba1*$^{f/f}$ and *Gba1*$^{f/f}$*::cre* mice (unpaired two-tailed Student's t-test; n = 5 mice per genotype).

Suppl. Fig. 7 Behavioral characterization of *Gba1*$^{f/f}$*::cre* mice. (a, b) Analysis of time spent in the external zone of the box during open field test of 6-month-old male (a) and female (b) *Gba1*$^{f/f}$ and *Gba1*$^{f/f}$*::cre* mice (unpaired two-tailed Student's t-test; *, $p < 0.05$; n = 9 *Gba1*$^{f/f}$ and n = 11 *Gba1*$^{f/f}$*::cre* mice). Error bars indicate s.e.m. **(c, d)** Rotarod performance of 6-month-old male (c) and female (d) *Gba1*$^{f/f}$ and *Gba1*$^{f/f}$*::cre* mice (unpaired two-tailed Student's t-test; n = 6 *Gba1*$^{f/f}$ and n = 10 *Gba1*$^{f/f}$*::cre* male mice and n = 6 *Gba1*$^{f/f}$ and n = 11 *Gba1*$^{f/f}$*::cre* female mice). Error bars indicate maximum and minimum values.

Supplementary figure 1

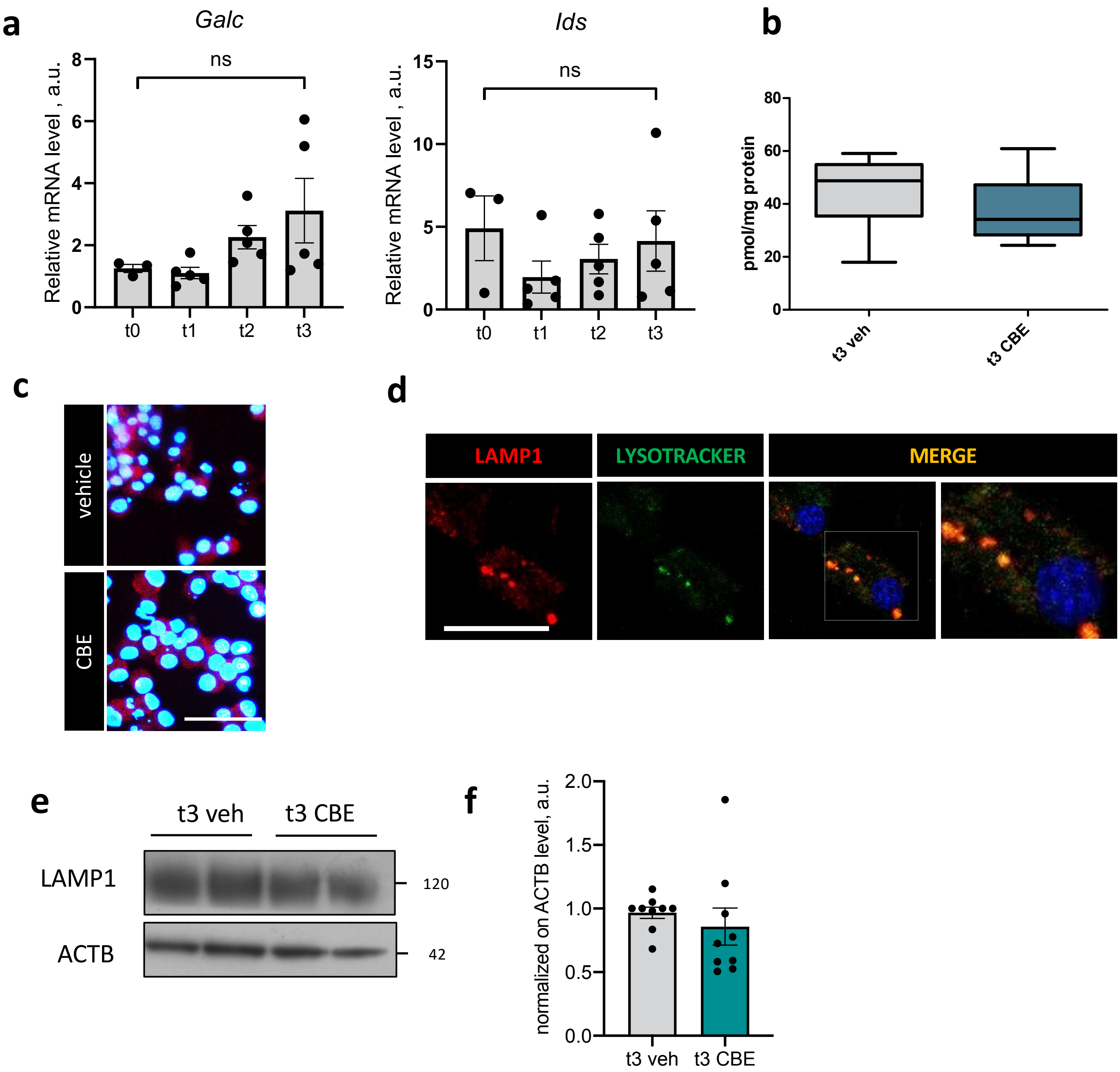

Supplementary figure 2

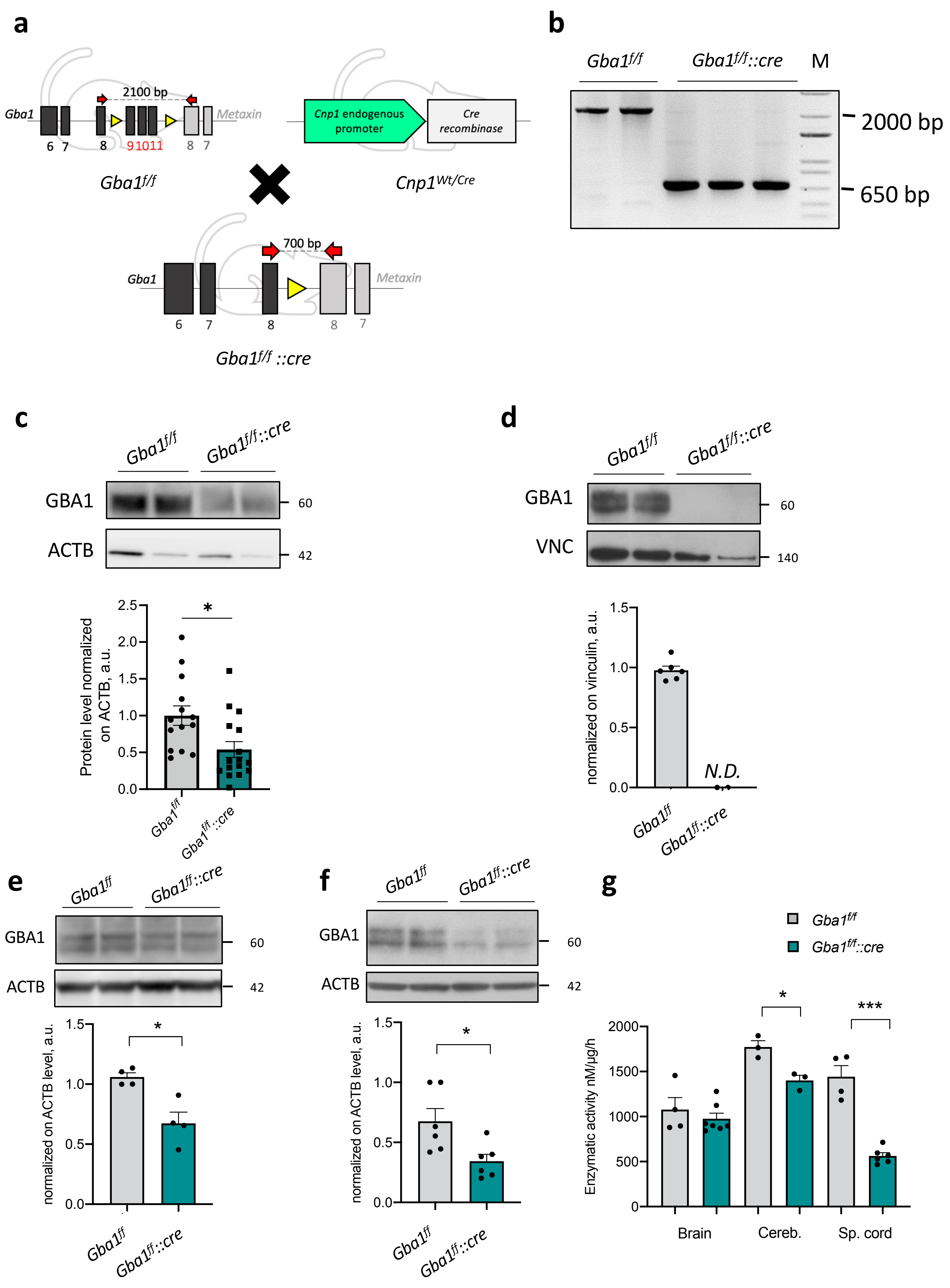

Supplementary figure 3

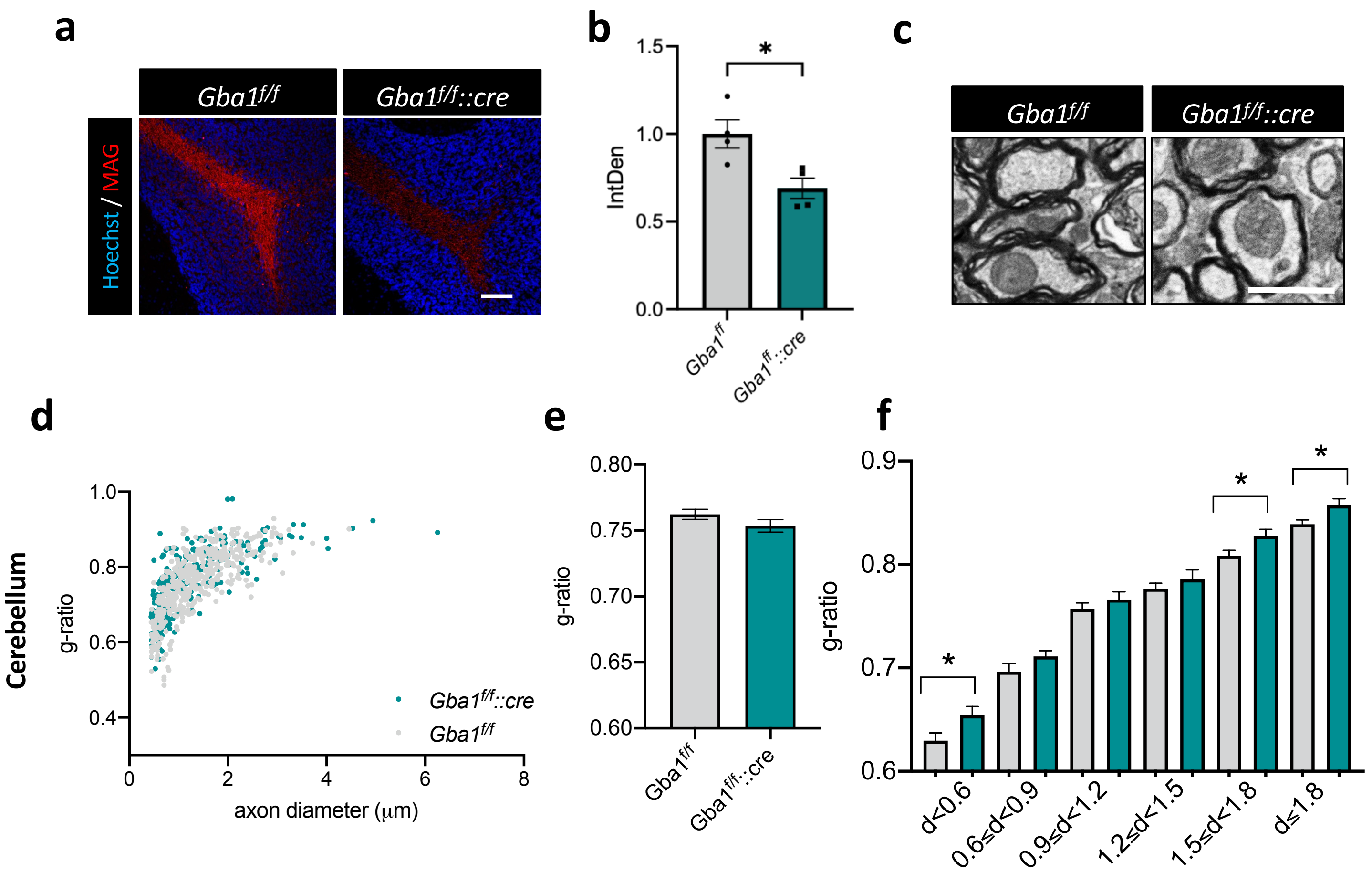

Supplementary figure 4

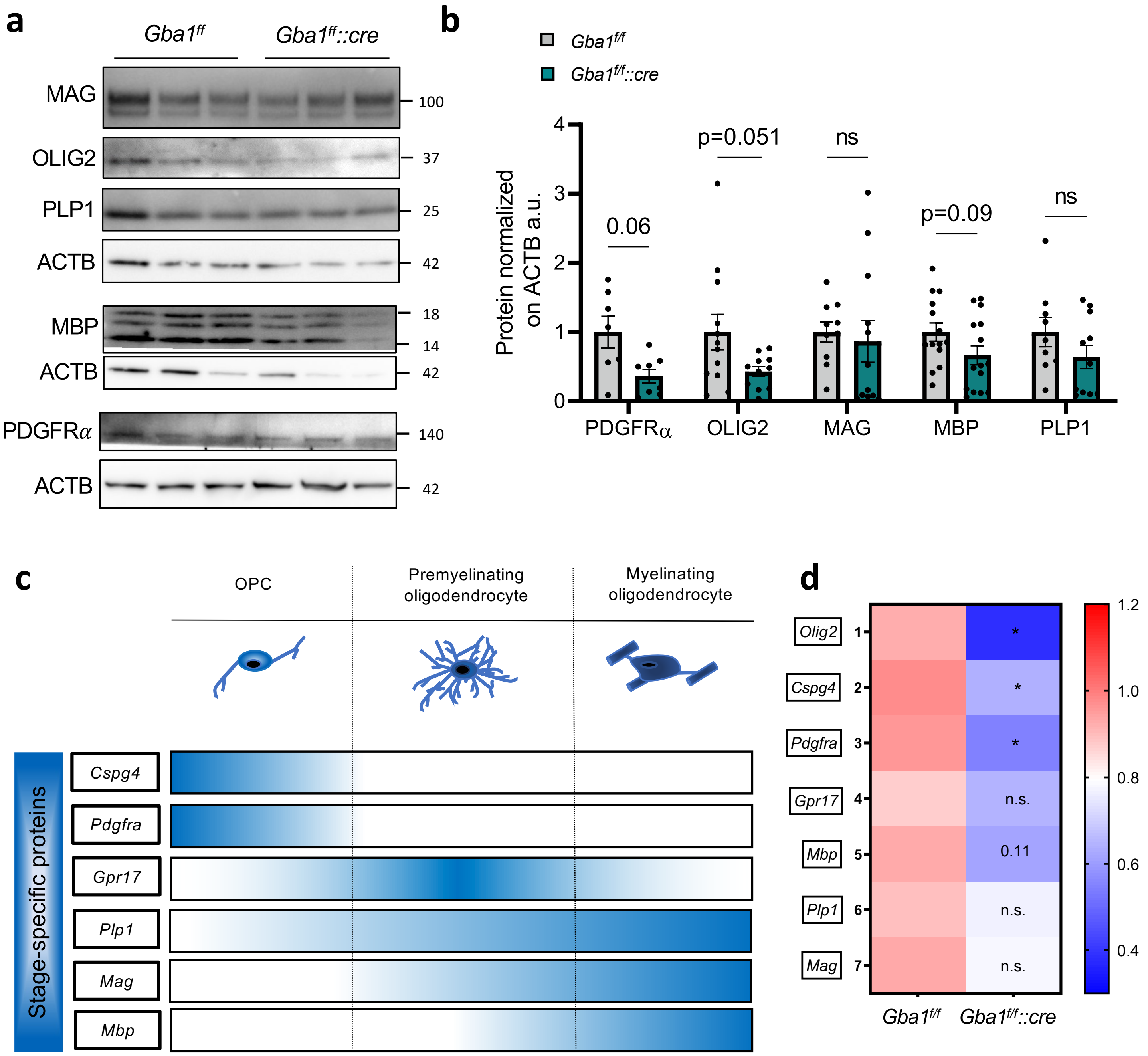

Supplementary figure 5

a

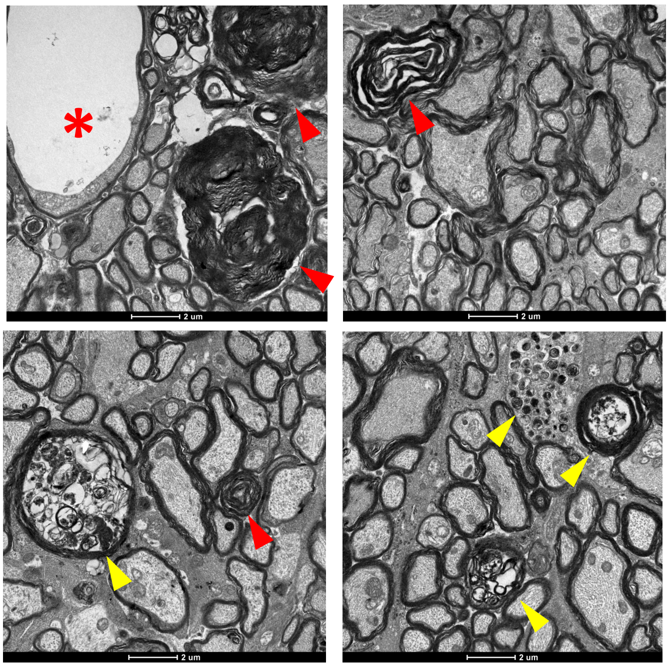

b

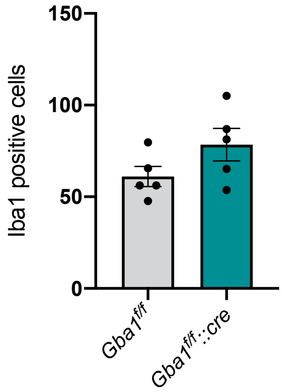

Supplementary figure 6

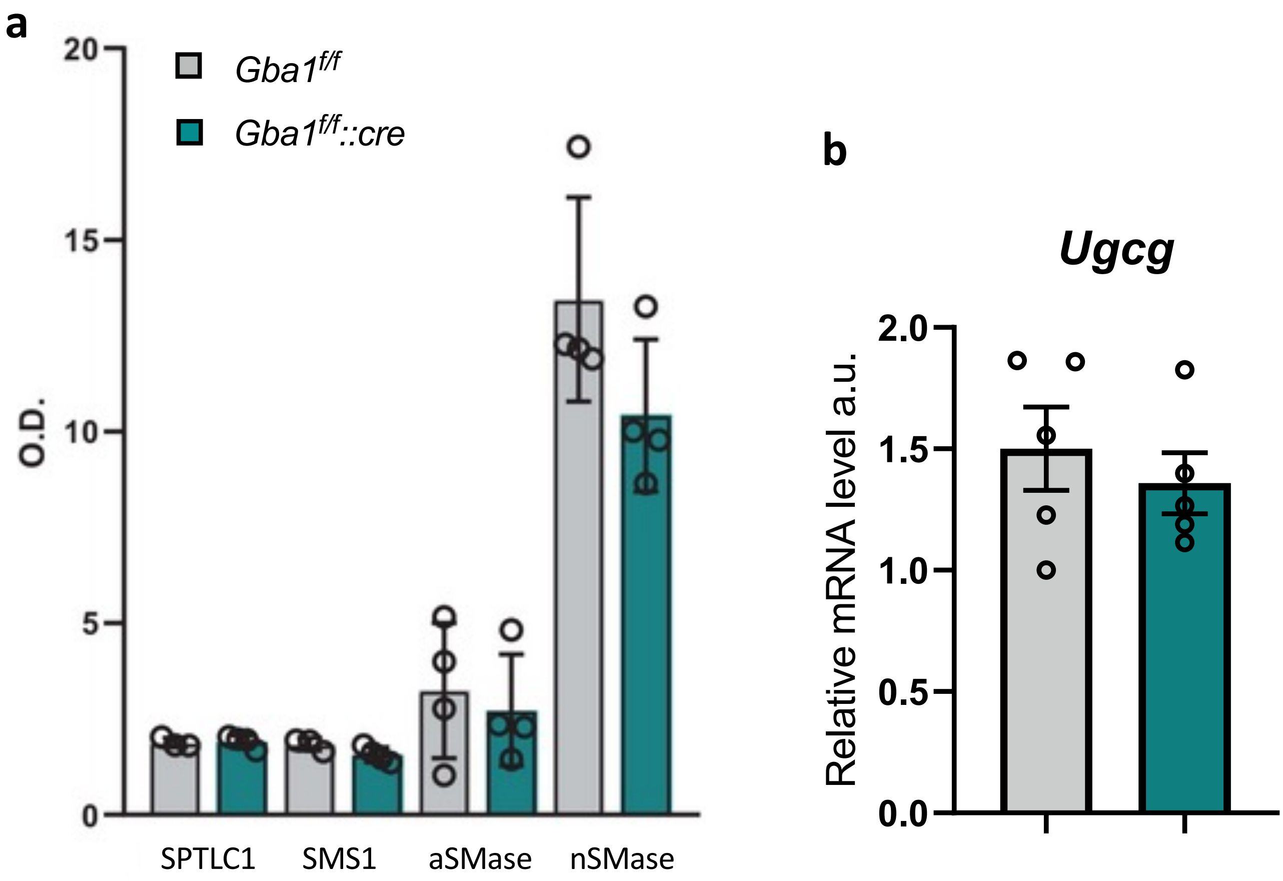

Supplementary figure 7

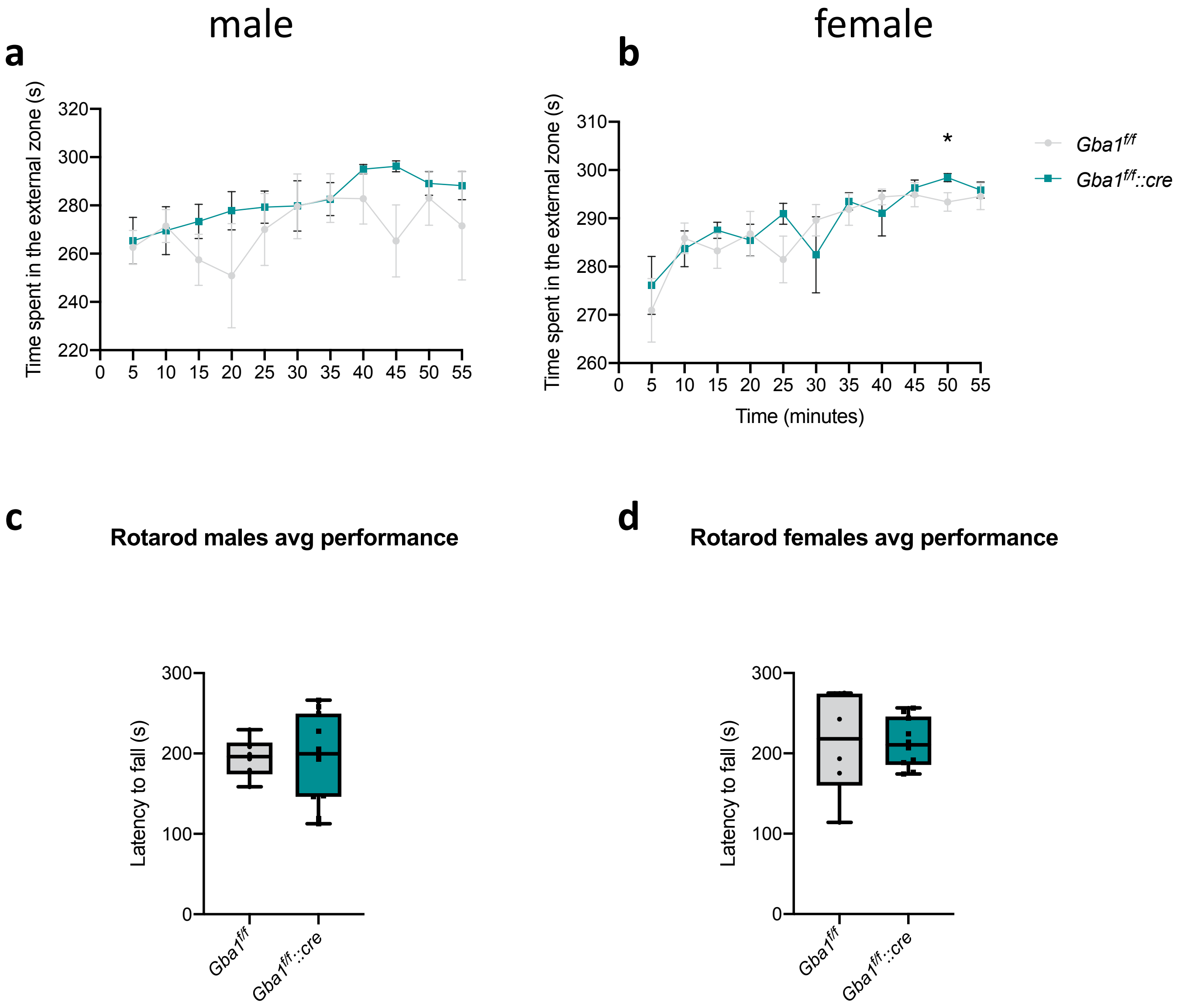

Supplement: Supplementary file 1 — Supplementary Material 1. [file 13024_2024_713_MOESM1_ESM.pdf]
